# Structure, gene composition, divergence time and phylogeny analysis of the woody desert species *Neltuma alba*, *Neltuma chilensis* and *Strombocarpa strombulifera*

**DOI:** 10.1038/s41598-024-64287-y

**Published:** 2024-06-13

**Authors:** Roberto Contreras-Díaz, Felipe S. Carevic, Liesbeth van den Brink, Wilson Huanca-Mamani, Patrick Jung

**Affiliations:** 1 Centro Regional de Investigación de Desarrollo Sustentable de Atacama (CRIDESAT), Universidad de Atacama, Copayapu 485, Copiapó, Chile; 2 Laboratorio de Ecología Vegetal, Facultad de Recursos Naturales Renovables, Universidad Arturo Prat, Campus Huayquique, Iquique, Chile; 3 Núcleo Milenio de Ecología Histórica Aplicada Para los Bosques Áridos (AFOREST), Santiago, Chile; 4 ECOBIOSIS, Departamento de Botánica, Facultad de Ciencias Naturales y Oceanográficas, Universidad de Concepción, Concepción, Chile; 5 Laboratorio de Biología Molecular de Plantas, Facultad de Ciencias Agronómicas, Centro de Genética y Genómica UASARA, Universidad de Tarapacá, 1000000 Arica, Chile; 6 Integrative Biotechnology, University of Applied Sciences Kaiserslautern, Carl-Schurz-Str. 10-16, 66953 Pirmasens, Germany

**Keywords:** Plant genetics, Ecology, Genetics

## Abstract

*Neltuma alba* (Algarrobo blanco), *Neltuma chilensis* (Algarrobo Chileno) and *Strombocarpa strombulifera* (Fortuna) are some of the few drought resistant trees and shrubs found in small highly fragmented populations, throughout the Atacama Desert. We reconstructed their plastid genomes using de novo assembly of paired-end reads from total genomic DNA. We found that the complete plastid genomes of *N. alba* and *N. chilensis* are larger in size compared to species of the *Strombocarpa* genus. The *Strombocarpa* species presented slightly more GC content than the *Neltuma* species. Therefore, we assume that *Strombocarpa* species have been exposed to stronger natural selection than *Neltuma* species. We observed high variation values in the number of cpSSRs (chloroplast simple sequence repeats) and repeated elements among *Neltuma* and *Strombocarpa* species. The p-distance results showed a low evolutionary divergence within the genus *Neltuma*, whereas a high evolutionary divergence was observed between *Strombocarpa* species. The molecular divergence time found in Neltuma and Strombocarpa show that these genera diverged in the late Oligocene. With this study we provide valuable information about tree species that provide important ecosystem services in hostile environments which can be used to determine these species in the geographically isolated communities, and keep the highly fragmented populations genetically healthy.

## Introduction

Legumes have a cosmopolitan distribution and are ecologically important in almost all biomes of the world as their fruits are a food source and their root biology nourishes soils^1,2^, even in ecosystems as extreme as the Atacama Desert. *Leguminosae* (*Fabaceae*) is one of the largest angiosperm family in terms of species numbers, one of the most diverse family and is classified into three subfamilies (*Caesalpinioideae*, *Mimosoideae*, and *Papilionoideae*), which have close to 770 genera and over 19,500 species^1,3^. The genus *Prosopis* L. (Mesquite) consisted of 57 species before the genus was disintegrated, which are distributed across Southwest Asia, Africa, and (predominantly) America^4–6^. *Prosopis* species from the *Strombocarpa* Bentham and *Algarobia* DC. Emend. Burk sections are trees occurring in arid and semiarid regions^5,7^. These species inhabit the Atacama Desert in Chile, which extends for over 1000 km between latitudes 19°S and 30°S and is bordered by the Coastal Cordillera in the west and the Andean Cordillera in the east^8^. Surviving in a place as hostile as the Atacama Desert, where radiation and water stress are high^9,10^ is already an accomplishment. However, these trees also provide local people with important resources such as fruits, juice, fodder and wood^7^. Three species from the formerly known *Strombocarpa* section (*Prosopis strombulifera* (Argentine screwbean) and the endemics *Prosopis burkartii* and *Prosopis tamarugo* (Tamarugo))^7,11^ and individuals belonging to different species from the formerly know *Algarobia* section (*Prosopis chilensis*, *Prosopis flexuosa*, and *Prosopis alba*) can be found in the Atacama Desert^5^. The scientific names of these species and the concept of *Prosopis* established by Bentham^12^ and Burkart^6^ has only currently been disintegrated, because *Prosopis* was found to be polyphyletic based on both chloroplast (cpDNA) and nuclear DNA (nDNA)^4,13,14^. As a consequence, the old *Prosopis* cluster was divided in six genera—*Anonychium*, *Prosopis*, *Neltuma*, *Strombocarpa*, *Xerocladia* and *Indopiptadenia*. The species of the above mentioned *Algarobia* section were renamed as *Neltuma chilensis*, *Neltuma flexuosa a*nd *Neltuma alba*, and the species of the above mentioned *Strombocarpa* section as *Strombocarpa strombulifera*, *Strombocarpa burkartii* and *Strombocarpa tamarugo*^4^. This division was based on short DNA sequences, as is common practice in taxonomy. However, compared to short DNA sequences, a complete plastid genome of approximately 160,000 bp can offer more information about the phylogenomic relationships and gives a full overview of the specific genes and the structure of its genome.

The plastid genome is a valuable taxonomic resource with rich genetic information^15^, as it is highly conserved and maternally inherited^16^. Because the plastid genome can provide valuable information to aid the conservation of threatened trees^17^, gaining insights in chloroplast DNA of the legume tree populations from Atacama Desert could help their conservation. Plastid genome sequences are commonly used in plant phylogeny, phylogeographic and genome evolution studies^15,16^. Lately, the use of complete plastid genome as a “super-barcoding” method has become an excellent approach allowing for the increase of the phylogenetic resolution at lower taxonomic levels in plants^18,19^. However, in the Atacama Desert only a few plastomes of the native and endemic herbaceous plants^20,21^, shrubs^22,23^ and leguminous trees^24,25^ have been characterized so far.

Unfortunately, several species of trees of the genera *Neltuma* and *Strombocarpa* are clasified in vulnerable and endangered conservation status in Chile, e.g. *Neltuma chilensis* and *Strombocarpa tamarugo*. *Neltuma chilensis* and *Neltuma alba* are restricted to southern Peru, northern and central Chile, southwestern Bolivia and northwestern, western and central Argentina^26–28^. The not threatened *Strombocarpa strombulifera* is widely distributed from the Arizona desert (U.S.A.) to Patagonia (Argentina)^29^. However, in the Atacama Desert, *Neltuma alba, Neltuma chilensis*, as well as *Strombocarpa strombulifera* populations, are fragmented and restricted to oases or valleys (forming populations of only a few individuals), and geographically isolated from each other by large areas of land^5,7,30^. This complicates the gene flow between these populations, decreasing their genetic diversity and therefore decreasing the chances to adapt to future environmental changes. As several species are morphologically hard to distinguish due to phenotypic plasticity, it is urgently necessary to identify the plastomes of their genera now, as the more endangered species might go extinct without proper management strategies. Until now, there is no complete plastid genome available for *N. alba*, *N. chilensis* and *S. strombulifera*. However, they are needed to confirm phylogenomic relationships between them, and with closely related species. In this study we provide and analyze the complete plastid genomes of *N. alba*, *N. chilensis* and *S. strombulifera*, in terms of structure, gene composition, divergence time and phylogeny.

## Methods

### Plant material and DNA isolation

Fresh leaves of *Neltuma alba* (Griseb.) C.E. Hughes & G.P. Lewis*, Neltuma chilensis* (Molina) C.E. Hughes & G.P. Lewis and *Strombocarpa strombulifera* (Lam.) A. Gray were collected in Copiapó (27°21′39.3ʺS 70°20′33.8ʺW), Chacabuco (33°05′24.9ʺS 70°39′07.3ʺW) and Pampa del Tamarugal (20°27′59.9ʺS 69°33′23.5ʺW) in Chile, respectively. Identification of samples was done according to the taxonomic criteria described by Burkart^6^. Additionally, the samples were verified by the forester Boris Burgos of the Corporación Nacional Forestal (CONAF) from the Atacama Region. The specimens were deposited in the Departamento de Silvicultura y Conservación de la Naturaleza herbarium of Universidad de Chile (under the names that were correct at the time of deposition: *Prosopis alba*, EIF13329; *Prosopis chilensis*, EIF13328; and *Prosopis strombulifera*, EIF13350). DNA was isolated from the leaves using the modified cetyl-trimethylammonium bromide (CTAB) protocol^7^. The DNA was quantified with a Qubit™ 3.0 fluorometer and a Qubit™ dsDNA HS Assay Kit, according to the protocol supplied by the manufacturer. DNA integrity was verified with an Agilent 2100 Bioanalyzer prior to sequencing.

### Genome sequencing, assembling and annotation

Sequencing libraries were generated by a TruSeq Nano DNA LT Kit (Illumina, San Diego, CA). The final libraries were run on an Agilent 2100 Bioanalyzer to verify the fragment size distribution and concentration. Sequencing was performed with an Illumina sequencing platform, at Genoma Mayor (Universidad Mayor, Chile). Paired-end sequences of 150 bp were generated for each read (R1 and R2). The filtered reads were assembled using SPAdes 4 software version 3.13.0^31^, using three k-mers parameters:—k 33, 55 and 77. The plastid was annotated with PGA software^32^ and CPGAVAS2^33^, after which it was manually corrected when needed. The graphical map of the plastid was generated by Organellar Genome DRAW (OGDRAW)^34^, and the complete nucleotide sequences were deposited in the NCBI GenBank database (OP672364, OP672365 and OP672366, under the names *Prosopis alba*, *Prosopis chilensis* and *Prosopis strombulifera*, respectively).

### Genome comparison, repeat and phylogenomic analysis

The plastid structures (LSC/IR, IR/SSC) of *N. alba*, *N. chilensis* and *S. strombulifera* and of five closely related species, i.e. *Neltuma juliflora* (Sw.) Raf., *Neltuma glandulosa* (Torr.) Britton & Rose, *Strombocarpa tamarugo* (Phil.) C.E. Hughes & G.P. Lewis, *Prosopis farcta* (Banks & Sol.) J.F. Macbr. and *Prosopis cineraria* (L.) Druce, of the *Mimoseae* tribe were visualized and compared using IRScope^35^. We used sequence data of whole plastomes (obained from GenBank) of species from the genera *Neltuma*, *Strombocarpa* and *Prosopis* for the identification of the simple sequence repeats (SSRs). These SSRs were identified using MISA software^36^ with the following search parameters: ten for mononucleotide, eight for dinucleotide, four for trinucleotide and tetranucleotide, and three for pentanucleotide and hexanucleotide. To identify the tandem repeats (forward, palindromic, reverse, and complement) of these species we used REPuter ^37^ with the following parameters: hamming distance equal to 3, minimal repeat size set to 30 bp, and maximum computed repeats set to 300 bp. The divergence among plastid genomes of *N. alba* and *N. chilensis*, *N. juliflora*, *N. pallida*, *N. glandulosa*, *S. tamarugo*, *S. strombulifera* and *P. farcta* was assessed using p-distance, and a second p-distance calculation was done to assess divergence between the focal species (mentioned before) and the rest species of the tribe *Mimoseae*, of which the plastid genome was available, using MEGA X^38^. The complete plastid genome sequence of *N. alba* (OP672364), *N. chilensis* (OP672365), *S. strombulifera* (OP672366), *S. tamarugo* (MW582314), *N. glandulosa* (NC_026683), *N. juliflora* (MN104889), *Neltuma pallida* (Humb. & Bonpl. ex Willd.) C.E.Hughes & G.P.Lewis (NC_084206), *P. farcta* (MZ073639), *P. cineraria* (MN104890), *Cylicodiscus gabunensis* Harms (MZ274089), *Entada phaseoloides* (L.) Merr. (NC_073582), *Piptadeniastrum africanum* (Hook.f.) Brenan. (MZ274093), *Xylia xylocarpa* (Roxb.) W.Theob. (NC_057267), *Adenanthera microsperma* Teijsm. & Binn. (NC_034986), *Dichrostachys cinerea* (L.) Wight & Arn. (NC_035346), *Mimosa bimucronata* (DC.) Kuntze (NC_061740), *Leucaena trichandra* (Zucc.) Urb (NC_028733), *Parkia timoriana* (DC.) Merr. (OK662459), *Parkia javanica* (Lam.) Merr. (NC_034989), *Piptadenia communis* Benth. (NC_034990), *Stryphnodendron adstringens* (Mart.) Coville (NC_044627), *Mimosa diplotricha* var. *intermis* (Adelb.) M.K. Alam & M. Yusof, *Mimosa pigra* L., and *Acacia ligulata* A.Cunn. ex Benth. (NC_045513) as outgroup species were used in the phylogenomic analysis. Seventy-six protein-coding genes (PCG) sequences were aligned separately using MAFFT v7^39^ and any gaps in the alignment were trimmed using TrimAL v1.4^40^. Afterwards, the sequences were concatenated with Mesquite 3.81 software^41^. The analyses of the 76 PCG sequences were conducted using the maximum likelihood (ML) method. The PCG sequences of the twenty four species were analyzed using the Bayesian inference (BI) methods. The best-fitting nucleotide substitution model of sequence evolution, model TVM + I + G, was determined based on the Akaike Information Criterion (AIC) using the MrModeltest v2.3^42^. The ML analyses were performed using RAxML-HPC BlackBox v.8.1.24^43^ with 1000 bootstrap replicates. The BI analysis was conducted using MrBayes v.3.2^44^ with the CIPRES Science Gateway v3.3^45^. The Markov Chain Monte Carlo (MCMC) algorithm was calculated for 5,000,000 generations, and the sampling tree for every 1,000 generations. The first 25% of generations were discarded as burn-in. In the analysis, bootstrap support (BS) values were estimated in the ML, and the reliability of clades in the Bayesian analysis was evaluated by means of posterior probability (PP). The trees were visualized with FigTree^46^.

### Divergence time estimate

To estimate the divergence time of the species we used BEAST v2.6.0^47^ based on the complete plastid genome sequence of eight species of the *Prosopis*, *Neltuma* and *Strombocarpa* genera, and *Leucaena trichandra* as an outgroup species. All genome sequences were aligned with MAFFT, and then the file was imported to BEAUTi interface to generate a file for BEAST, after applying HKY + Γ substitution model, “Empirical” frequency, strict molecular clock model and “Yule” model speciation. Divergence times were estimated combining two calibration points. The TimeTree tool (http://www.timetree.org^48^) was used to fix the node age of *Leucaena trichandra* and the *Prosopis* genus, which is known to have diverged 35 Mya (33.2–40.3 Mya)^13,49–51^. We considered a second calibration as well, fixing the node age of *Series Pallidae, Chilenses, and Ruscifoliae* clade (*N. alba*, *N. chilensis*, *N. juliflora* and *N. pallida*, among others) at 3.65 Mya (3.31–3.99 Mya)^13^. The Markov Chain Monte Carlo (MCMC) was run for 6 million generations, sampling every 1,000 generations. We ran the program using the input file XML generated by BEAUTi in BEAST. Final log files were checked in Tracer 1.7.1^52^. We used the TreeAnnotator program^47^ with a 10% burn-in. Phylogenetic trees were summarized by Figtree software, using the extent of the 95% highest posterior density (HPD) intervals for each divergence time. The geological timescale was calculated with the strap palcakge geoscalePhylo in R^53^. Additionally, we complemented this analysis using DnaSP v. 5 software^54^ and manually explored alignments for changes with MEGA X^38^: i.e. in the protein coding region, upstream or downstream, identifying the presence of pseudogenes, disrupted genes within the reading frames or indels.

### Ethics approval

This article does not contain any studies with human participants or animals performed by any of the authors.

### Research permit

This research complies with the corresponding research permits according to national and international standards, for the collection of material from *Neltuma alba*, *Neltuma chilensis* and *Strombocarpa strombulifera*, and the care of flora and fauna. The research permit was granted by CONAF (National Forestry Corporation) N° N00024/08-11-2019 (JBH/FAP/JVO) and N° N00003-2023/27-01-2023 (NOO/FAP/JVO).

### Consent to participate

All the authors of this manuscript declare that we participated in the design and preparation of this manuscript.

## Results

A total of 12,339,246; 10,766,088 and 10, 071,214 raw Illumina paired-end reads (150 bp) from *N. alba*, *N. chilensis* and *S. strombulifera* genomes were generated, respectively. After trimming adapters and low-quality bases, a total of ~ 10,000,000 reads for each species were used to assemble the plastid genome. The de novo assembly generated 245,005 contigs with an N50 length of 1740 bp and a total length of 342.2 Mb for *N. alba*, 237,706 contigs with an N50 length of 1674 bp and a total length of 324.8 Mb for *N. chilensis*, and 188,597 contigs with an N50 length of 2104 bp and a total length of 298.5 Mb for *S. strombulifera*.

**Figure 1:**
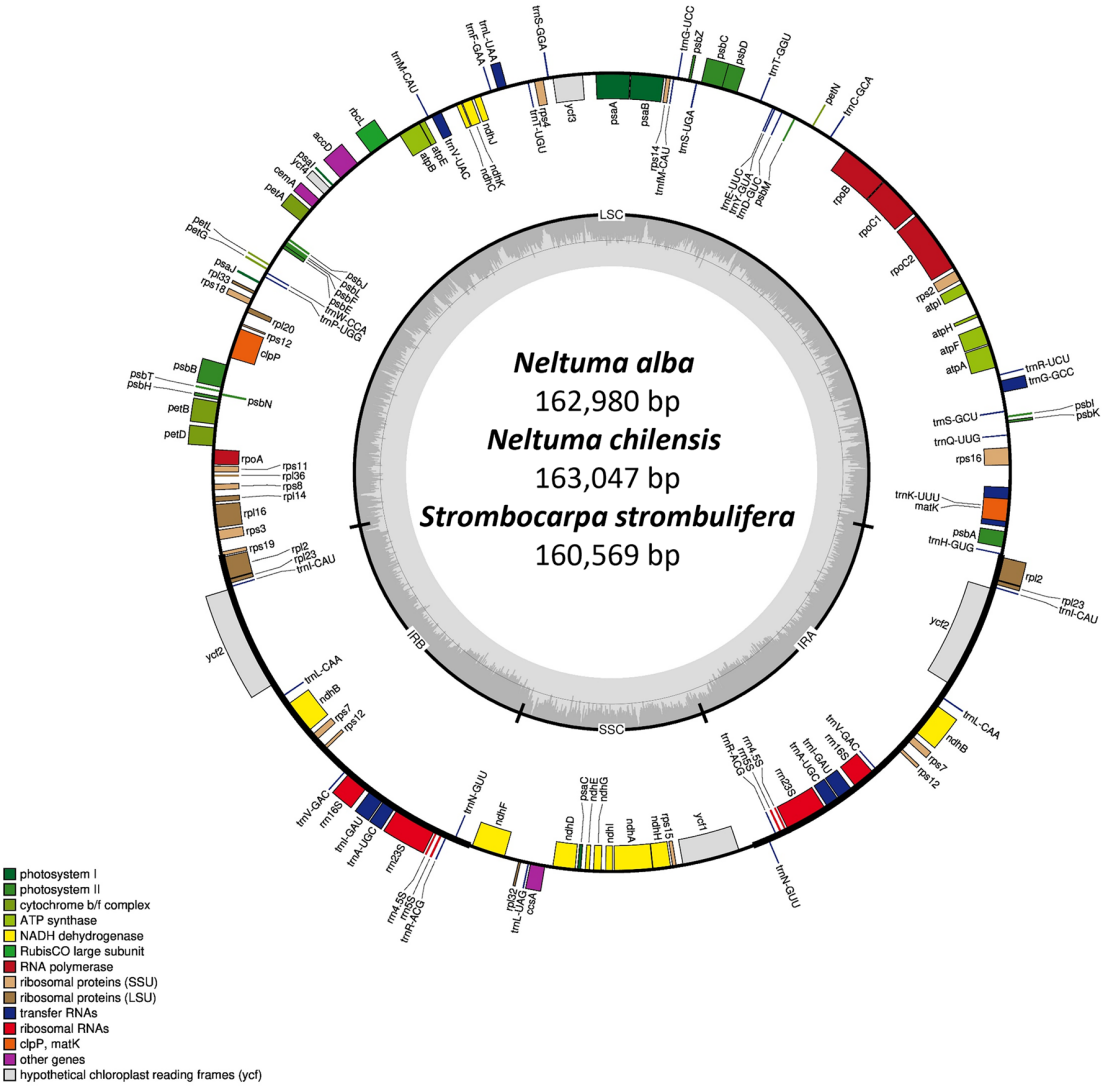
Circular gene map of the plastid genomes of *Neltuma alba*, *Neltuma chilensis* and *Strombocarpa strombulifera*. Genes were colored according to their functional group. Small single copy (SSC), large single copy (LSC), and inverted repeats (IRA, IRB) were indicated.

The plastid genome lengths of *N. alba*, *N. chilensis* and *S. strombulifera* comprise 162,980 bp, 163,047 bp and 160,569 bp, respectively and its structure contains a typical quadripartite structure with two inverted repeat regions (IR; 25,919 bp, 25,919 bp and 26,026 bp respectively) separated by a large single copy region (LSC; 92,300 bp, 92,356 bp and 89,569 bp respectively) and a small single copy region (SSC; 18,842 bp, 18,853 bp and 18,623 bp respectively) (Fig. 1, Table 1). Its length and structure are similar to those of the other species of the *Neltuma*, *Strombocarpa* and *Prosopis* genera, which vary in the IRs between 25,919 bp and 25,935 bp, in the LSC between 91,062 bp and 92,937 bp and in the SSC between 18,643 bp and 18,880 bp (Table 1). The cp genomes of the *Strombocarpa* species (*S. strombulifera* 160,569 bp; and *S. tamarugo* 161,575) are smaller (~ 806 to 2478 bp) than those of the *Neltuma* species (*N. pallida* 162,381; and *N. chilensis* 163,047) (Table 1). The GC content in the chloroplast of *N. alba* and *N. chilensis* (35.9%) is slightly less than in *S. strombulifera* (36.2%), but the overall GC content was similar to other species of the *Neltuma*, *Strombocarpa* and *Prosopis* genera (Table 1).

**Table 1:** General features of the *Neltuma*, *Strombocarpa* and *Prosopis* plastid genomes.

Species	Accession	Size (bp)	GC (%)	LSC (bp)	SSC (bp)	IR (bp)	N° genes	Protein-coding genes	tRNA genes	rRNA genes	References
*N. alba*	OP672364	162,980	35.9	92,300	18,842	25,919	129	84	37	8	This study
*N. chilensis*	OP672365	163,047	35.9	92,356	18,853	25,919	129	84	37	8	This study
*N. glandulosa*	NC_026683	163,040	35.9	92,322	18,880	25,919	128	83	37	8	Unpublished
*N. juliflora*	MN104889	163,237	35.9	92,495	18,880	25,931	132	85	39	8	Asaf et al. 2020^60^
*N. pallida*	NC_084206	162,381	35.9	91,805	18,748	25,914	132	85	39	8	Caycho et al. 2023^55^
*S. strombulifera*	OP672366	160,569	36.2	89,569	18,623	26,026	128	83	37	8	This study
*S. tamarugo*	MW582314	161,575	36.0	91,062	18,643	25,935	128	83	37	8	Contreras et al. 2021^24,28^
P. cineraria*	MN104890	163,677	35.9	92,937	18,878	25,931	131	85	38	8	Asaf et al. 2020^60^
*P. farcta*	MZ073639	162,900	35.9	92,156	18,880	25,932	127	82	37	8	Unpublished

In total, 128–129 genes were found in cp genomes of *N. alba*, *N. chilensis* and *S. strombulifera*, which included 83–84 coding genes, 8 rRNA genes, and 37 tRNA genes (Table 1). Of these genes, six coding genes (*ndhB*, *rpl2*, *rpl23*, *rps12*, *rps7* and *ycf2*), four rRNA genes and seven tRNA genes located in the IR regions contained duplicated genes (Table 2). Eighteen genes had introns, including nine PCGs (*atpF*, *ndhA*, *ndhB*, *petB*, *petD*, *rpl2*, *rpl16*, *rpoC1* and *rps16*), except for *S. strombulifera*, which had ten PCGs, additionally including *ycf2* to the ones mentioned before. Six tRNAs (*trnA*^*UGC*^, *trnG*^*GCC*^, *trnI*^*GAU*^, *trnK*^*UUU*^, *trnL*^*UAA*^ and *trnV*^*UAC*^) contained one intron, while three PCGs (*ycf3*, *rps12* and *clpP*) had two introns (Table 2).

**Table 2:** Gene composition of the plastid genome of *N. alba, N. chilensis and S. strombulifera*.

Category of genes	Group of genes	Names of genes	
Photosynthesis	Photosystem I	*psaA, psaB, psaC, psaI, psaJ*	
	Photosystem II	*psbA, psbB, psbC, psbD, psbE, psbF, psbH, psbI, psbJ, psbK, psbL, psbM, psbN, psbT, psbZ*	
	ATP synthase	*atpA, atpB, atpE, atpF*^*b*^*, atpH, atpI*	
	NADH-dehydrogenase	*ndhA*^*b*^*, ndhB*^*ab*^*, ndhC, ndhD, ndhE, ndhF, ndhG, ndhH, ndhI, ndhJ, ndhK*	
	cytochrome b/f complex	*petA, petB*^*b*^*, petD*^*b*^*, petG, petL, petN*	
	Large subunit RUBISCO	*rbcL*	
Protein synthesis and DNA replication	Transfer RNAs	*trnA-UGC*^*ab*^*, trnC-GCA, trnD-GUC, trnE-UUC, trnF-GAA, trnfM-CAU, trnG-UCC, trnG-GCC*^*b*^*, trnH-GUG, trnI-GAU*^*ab*^*, trnI-CAU*^*a*^*, trnK-UUU*^*b*^*, trnL-UAA*^*b*^*, trnL-CAA*^*a*^*, trnL-UAG, trnM-CAU, trnN-GUU*^*a*^*, trnP-UGG, trnQ-UUG, trnR-ACG*^*a*^*, trnR-UCU, trnS-GGA, trnS-UGA, trnS-GCU, trnT-GGU, trnT-UGU, trnV-UAC*^*b*^*, trnV-GAC*^*a*^*, trnW-CCA, trnY-GUA*	
	Ribosomal RNAs	*rrn16S*^*a*^*, rrn23S*^*a*^*, rrn4.5S*^*a*^*, rrn5S*^*a*^	
	Ribosomal Protein large-subunit	*rpl14, rpl2*^*ab*^*, rpl16*^*b*^*, rpl20, rpl23*^*a*^*, rpl32, rpl33, rpl36*	
	DNA dependent RNA polymerase	*rpoA, rpoB, rpoC1*^*b*^*, rpoC2*	
	Ribosomal Protein Small-subunit	*rps11, rps12*^*ac*^*, rps14, rps15, rps16*^*b*^*, rps18, rps19, rps2, rps3, rps4, rps7*^*a*^*, rps8*	
Other functions	Subunit of Acetyl-CoA-carboxylase	*accD*	
	c-type cytochrome synthesis gene	*ccsA*	
	Envelop membrane protein	*cemA*	
	Protease	*clpP *^*c*^	
	Maturase	*matK*	
	Initiation Factor	*infA*	
Unknown function	Conserved open reading frames	*ycf1, ycf2*^*a*^*, ycf3 *^*c*^*, ycf4*	

^a^Duplicated genes; ^b^Genes containing one intron; ^c^Genes containing two introns.

**Figure 2:**
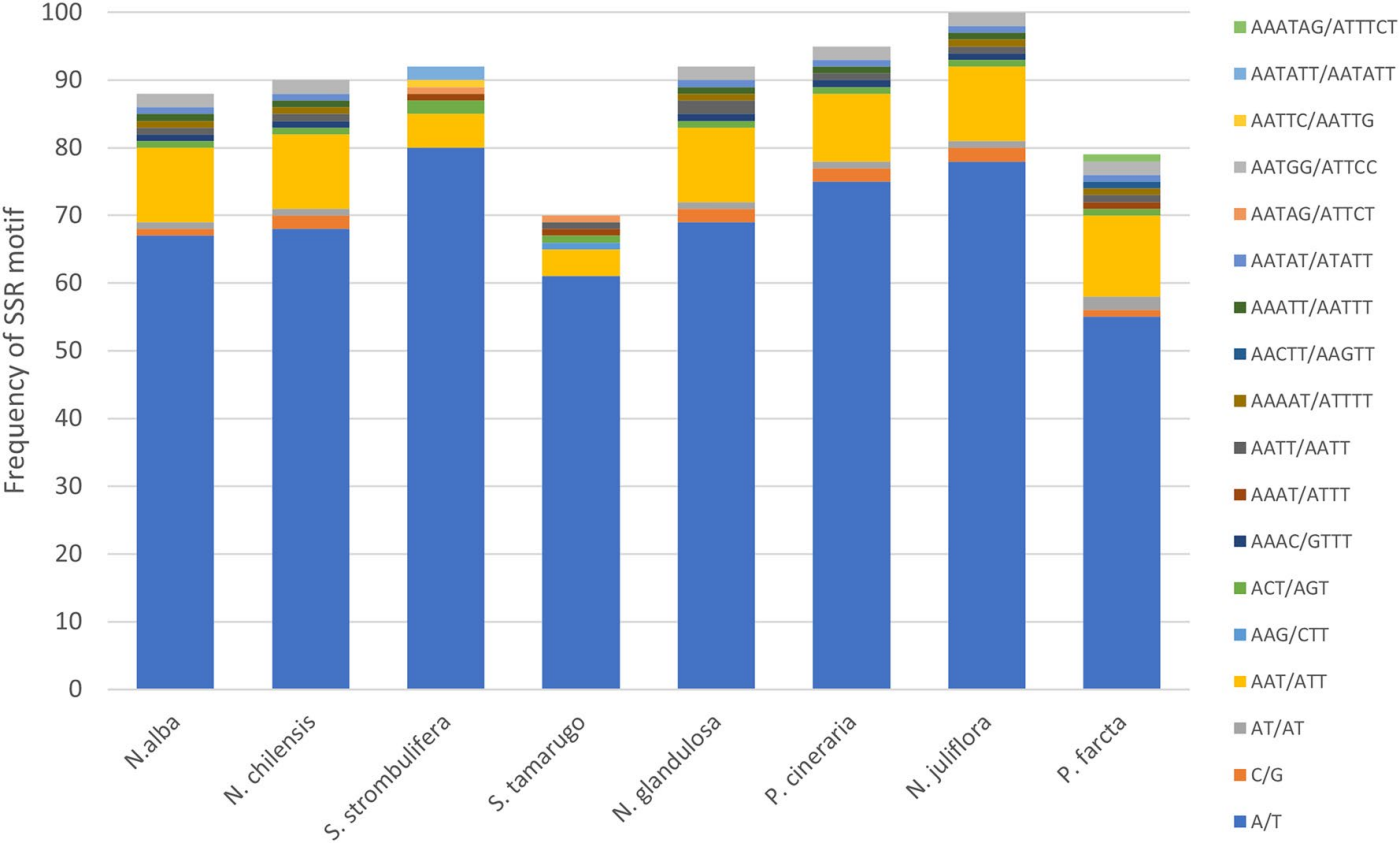
Frequency of SSR motifs in different repeat class types of the *N. alba*, *N. chilensis*, *S. strombulifera*, *S. tamarugo*, *N. glandulosa*, *P. cineraria*, *N. juliflora* and *P. farcta* plastid genomes.

We compared the simple sequence repeats (SSRs) from the cp genomes of the species of the *Neltuma*, *Strombocarpa* and *Prosopis* genera. The maximum number was 100 SSRs in *N. juliflora*, followed by 95 SSRs in *P. cineraria*, 92 SSRs in *N. glandulosa* and *S. strombulifera*, 90 SSRs in *N. chilensis*, 88 SSRs in *N. alba* and, whereas S. *tamarugo* and *P. farcta* only had 70 SSRs (Fig. 2, supplementary information: Fig. S1). Mononucleotide A/T repeats were the most common repeats, ranging from 61 (*S. tamarugo*) to 80 (*S. strombulifera*); mononucleotide C/G repeats were found in all species except in *S. tamarugo* and *S. strombulifera* (Fig. 2). The number of dinucleotide SSRs (AT/AT) was similar in all species except in *S. tamarugo* and *S. strombulifera* in which no dinucleotide SSRs were found. In all species, 10–12 trinucleotide repeats AAT/ATT were found, but in *S. tamarugo* and *S. strombulifera* only 4 and 5, respectively, were present. Only *S. tamarugo* had trinucleotide repeats AAG/CTT (Fig. 2). In general, four pentanucleotide SSRs type (AAAAT/ATTTT, AAATT/AATTT, AATAT/ATATT and AATGG/ATTCC) were found in all species, but only one pentanucleotide SSR was present in *P. farcta* (AACTT/AAGTT), *S. strombulifera* and *S. tamarugo* (AATAG/ATTCT), and *S. strombulifera* (AATTC/AATTG) (Fig. 2). Only two hexanucleotide SSRs, AATATT/AATATT and AAATAG/ATTTCT, were observed in *S. strombulifera* and *P. farcta*, respectively (Fig. 2).

**Figure 3:**
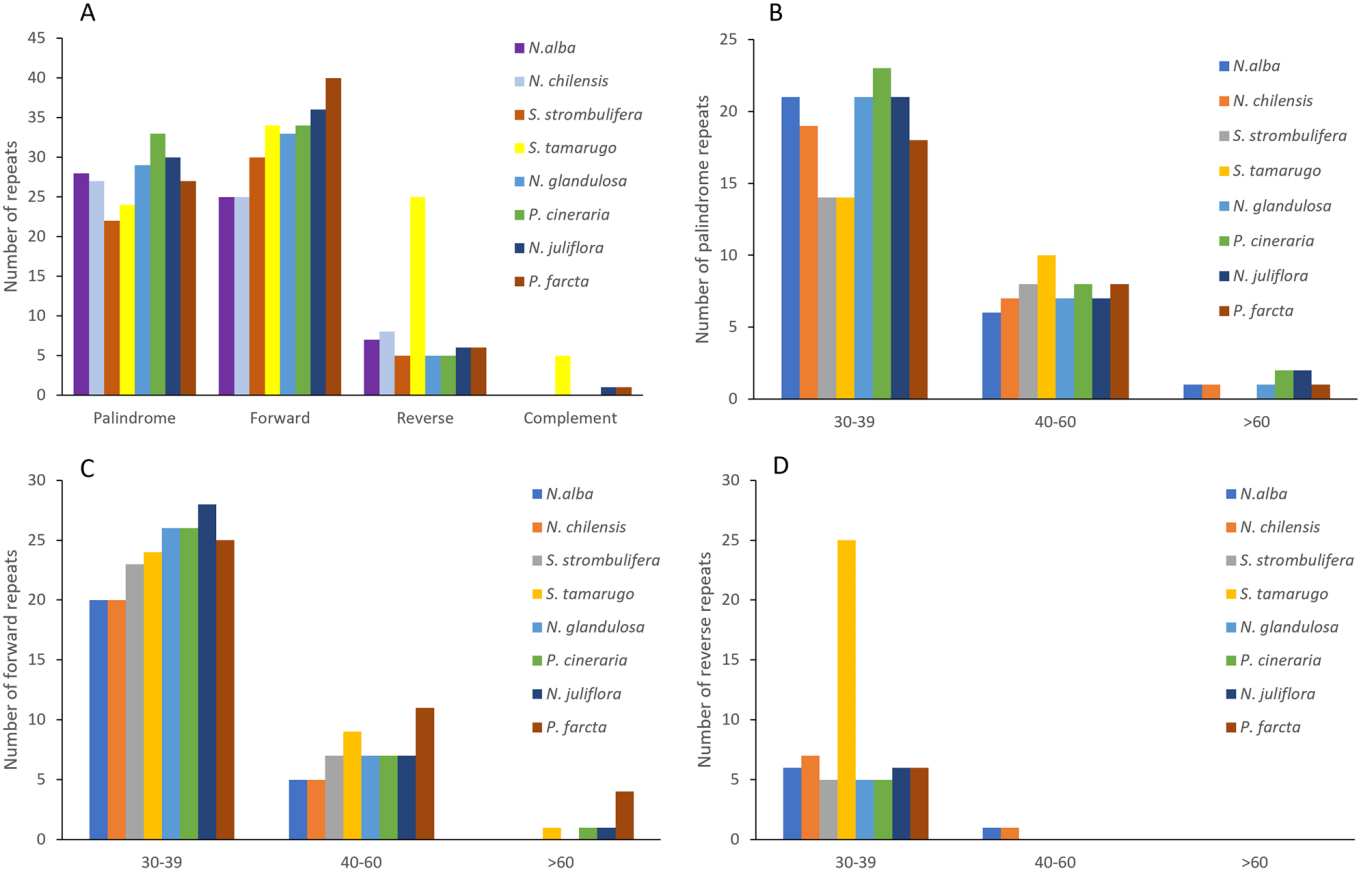
Repeat structure analysis of the *N. alba*, *N. chilensis*, *S. strombulifera*, *S. tamarugo*, *N. glandulosa*, *N. juliflora*, *P. cineraria* and *P. farcta* plastid genomes. Total numbers long repeat types: Palindrome, Forward, Reverse and Complement (**A**), number of palindrome repeats (**B**), number of forward repeats (**C**) and number of reverse repeats (**D**) by length.

The amounts of repeats varied between 57 and 88 in the cp genomes of *S. tamarugo* (88), *P. farcta* (74), *N. juliflora* (73), *P. cineraria* (72), *N. glandulosa* (67), *N. alba* (60), *N. chilensis* (60) and *S. strombulifera* (57) (Fig. 3A). The number of complement repeat in *S. tamarugo* was higher (5 repeats) than in the rest of the species (Fig. 3A). The total number of palindromic repeats was less in *Strombocarpa* species (*S. strombulifera*, 22; and *S. tamarugo*, 24) than in species of the *Neltuma* (*N. alba*, 28; *N. chilensis*, 27; *N. glandulosa*, 29; and *N. juliflora*, 30) and *Prosopis* (*P. cineraria*, 33; *P. farcta*, 27) genera (Fig. 3B). On the other hand, the total number of forward repeats was less in the cp genome of *N. alba* and *N. chilensis* (25 each), than in the rest of the species, where they varied between 30 to 40 (Fig. 3C). Palindromic and forward repeats with lengths of 30–39 bp were the most common and abundant repeats in the species of the *Neltuma*, *Strombocarpa* and *Prosopis* genera (Fig. 3B,C). The number of reverse repeat (range of 30–39 bp) in *S. tamarugo* was higher (with 25 repeats) than in the rest of the species (Fig. 3D).

**Figure 4:**
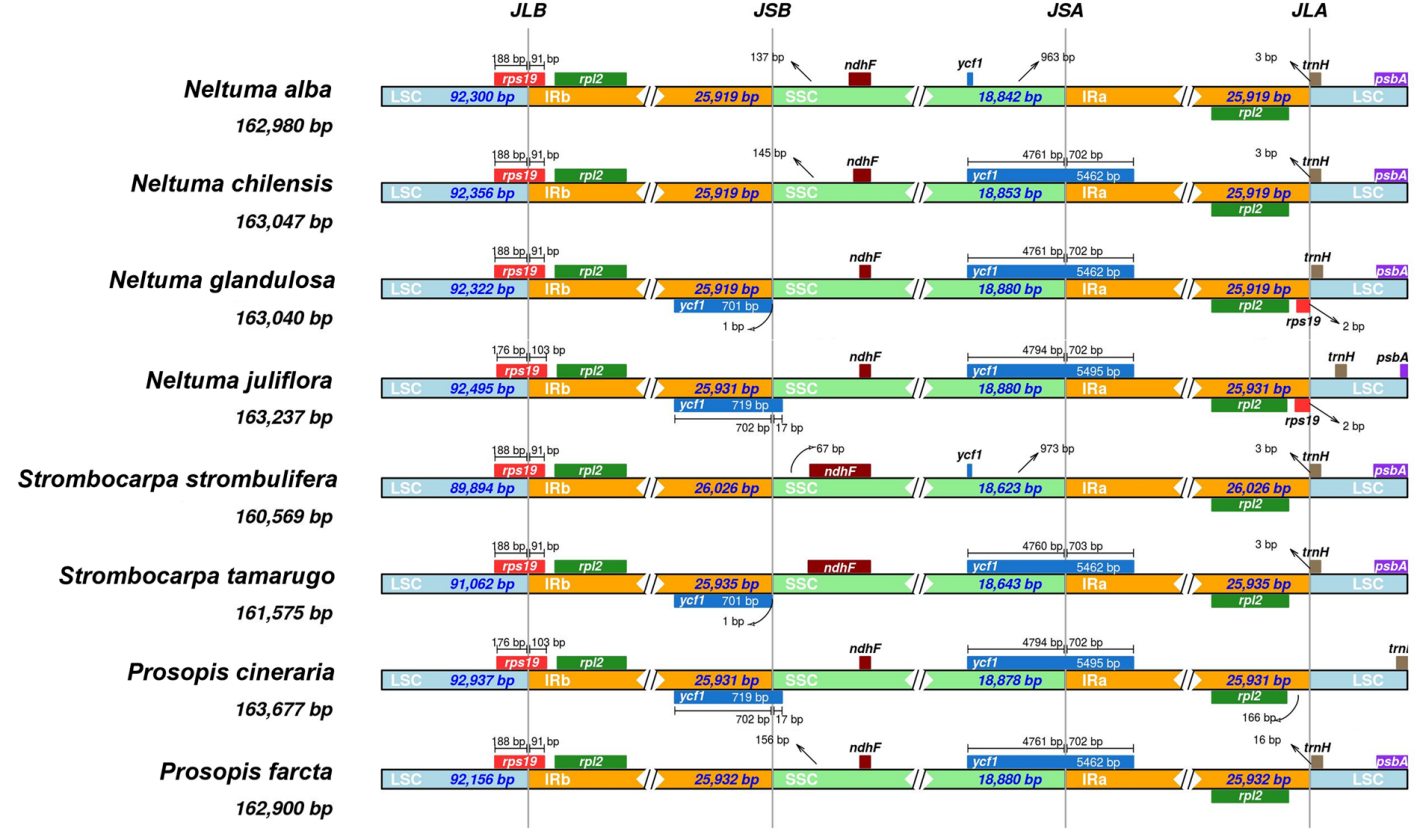
Comparison of plastid genomes between the Long Single Copy (LSC), Short Single Copy (SSC) and Inverted Repeat (IRa and IRb) junction regions among *Neltuma*, *Strombocarpa* and *Prosopis* species.

The expansion and contraction of the IR and SC regions contributes to the differentiation in plastid genome size in some genera and families. For that reason, we compared the SSC, LSC, IRa, and IRb border regions of the species of the *Neltuma*, *Strombocarpa* and *Prosopis* genera. In all species, *rps19* genes were located in the junction between LSC and IRb region (JLB), of which 176 to 188 bp were located at the LSC region and 91 to 103 bp located at the IRb region (Fig. 4). In all species, the *rpl2* gene was entirely located in the IR regions (Fig. 4). The *ndhF* gene of *Neltuma* and *Prosopis* species were located in the SSC region, 137–156 bp away from the IRb-SSC border, while in the two *Strombocarpa* species this gene was located approx. 67 bp away from the IRb-SSC border. At the SSC-IRa border, the *ycf1* gene extended into the SSC region, at varying lengths ranging from 4760 bp in *S. tamarugo* to 4794 bp in *N. juliflora* and *P. cineraria*, however, in *N. alba* and *S. strombulifera* the gene was entirely located in the SSC region, 963–973 bp away from the IRa-SSC border. In general, the truncated copy of *ycf1* was located in the IRb region (except in *N. alba* and *S. strombulifera*), while one end extended into the SSC region for 17 bp only in *N. juliflora* and *P. cineraria*. The distance between *rps19* and the IRa-LSC border was only 2 bp in *N. glandulosa* and *N. juliflora*. In most species, the *trnH* gene was located in the LSC region, 2–16 bp away from the IRa-LSC border, but in *N. juliflora* and *P. cineraria* it was much more distant. In general, the structure of the cp genomes of *Neltuma*, *Strombocarpa* and *Prosopis* were similar in arrangement (Fig. 4).

**Figure 5:**
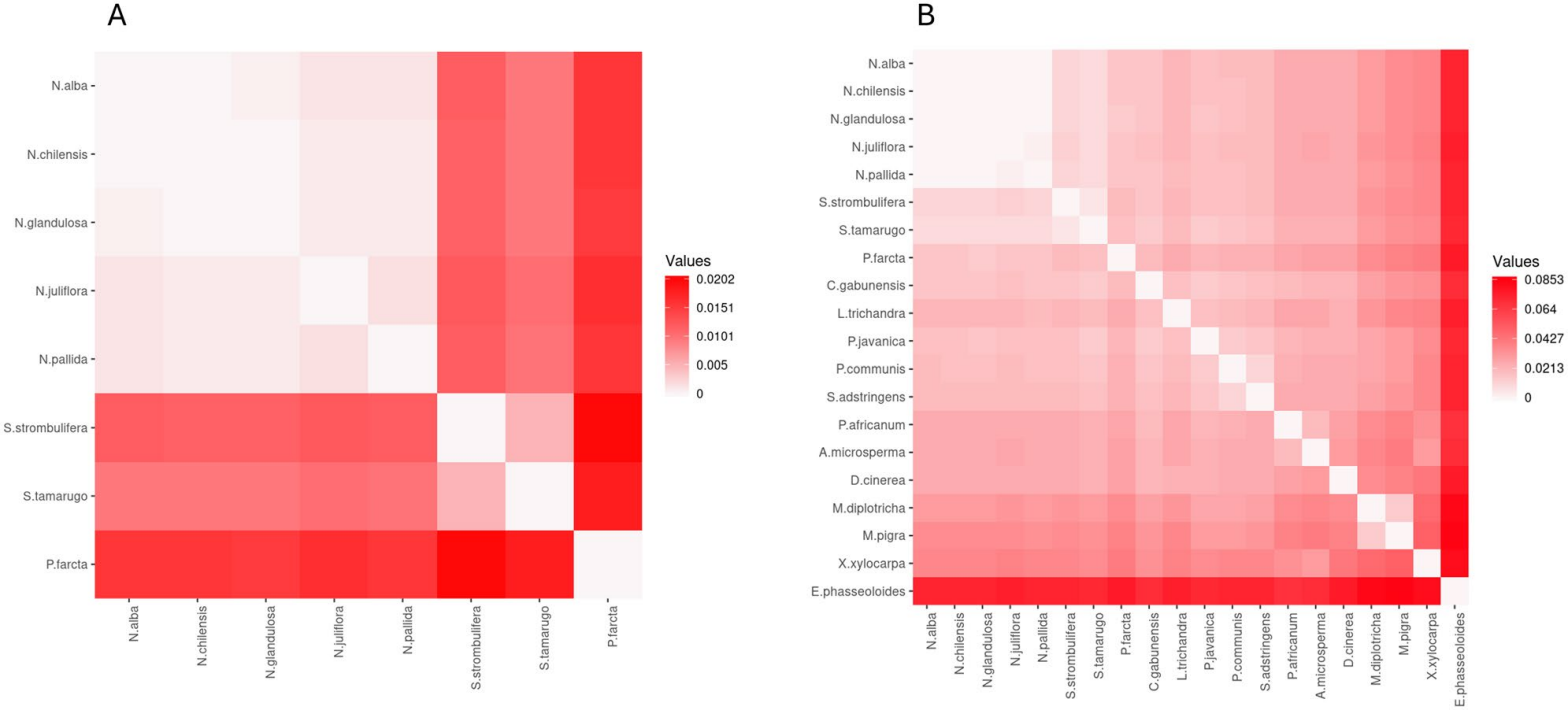
Evolutionary divergence heatmap of the plastid genomes of the *Neltuma*, *Strombocarpa* and *Prosopis* species (**A**), and twenty species of the tribe *Mimoseae* (**B**). p-distance value is indicated in the legends of the respective plots.

The mean p-distance among *Neltuma*, *Strombocarpa* and *Prosopis* species was 0.008543 with the lowest divergence (0.000295) between *N. chilensis* and *N. glandulosa*, and the largest divergence (0.020162) between *P. farcta* and *S. strombulifera* (Fig. 5A, Supplementary information: Table S1). The average evolutionary divergence was 0.00522 between Strombocarpa species, and 0.00100 among Neltuma species (Supplementary information: Table S1). Additionally, the overall sequence divergence, estimated by p-distance among the 20 plastid genome of Mimoseae, was 0.028122; the largest sequence divergence was observed between *E. phaseoloides* and *M. pigra* (0.085305) and the lowest divergence was, again, between *N. chilensis* and *N. glandulosa* (Fig. 5B, Supplementary information: Table S2).

**Figure 6:**
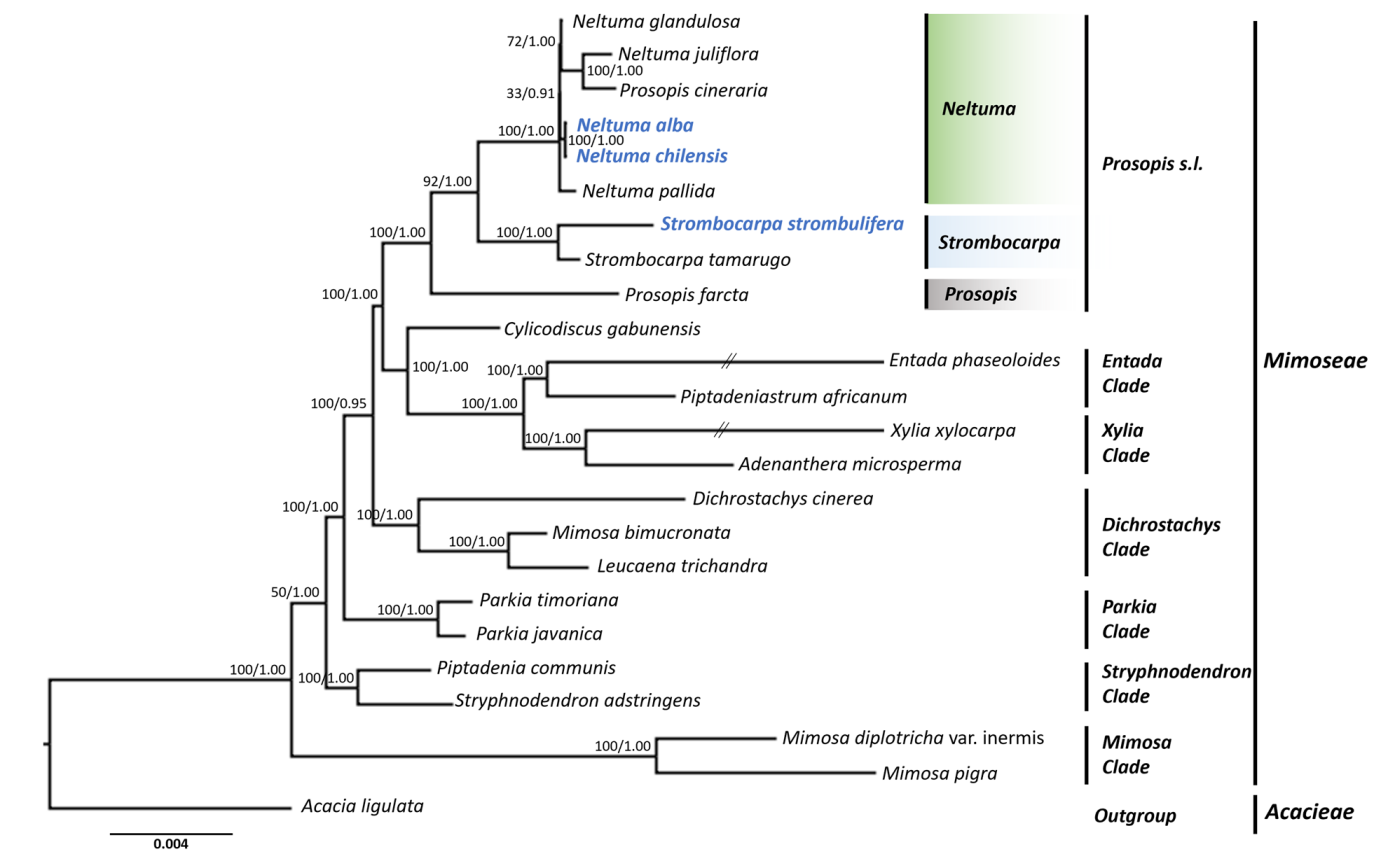
Molecular phylogenomic analysis based on 76 protein-coding genes of the plastid genome of 23 *Mimoseae* species and one *Acacieae* species as outgroup inferred by maximum likelihood and Bayesian inference methods. Numbers in the nodes are bootstrap support (BS) / posterior probabilities (PP).

The ML and BI phylogenetic analyses had similar topologies when we compared 76 protein coding genes of the plastid genomes (Fig. 6). The phylogenomic analysis revealed seven clades and one outgroup species *A. ligulata*. The clade *Prosopis* s.l. was divided into three well-supported subclades (BS = 100; PP = 1.00), formed by *Prosopis*, *Strombocarpa* and *Neltuma*. Of the three subclades, one subclade *Prosopis* consists of *P. farcta*; the second subclade *Strombocarpa*, includes *S. strombulifera* and *S. tamarugo* (BS = 100; PP = 1.00); and the third subclade *Neltuma* (BS = 100; PP = 1.00) is formed by *N. pallida*, *N. glandulosa*, *N. chilensis*, *N. alba*, *N. juliflora* and *P. cineraria*. Subsequently, we have six well supported main clades (BS = 100; PP = 1.00) formed by *Entada*, *Xylia*, *Dichrostachys*, *Parkia*, *Stryphnodendron* and *Mimosa* species (Fig. 6).

**Figure 7:**
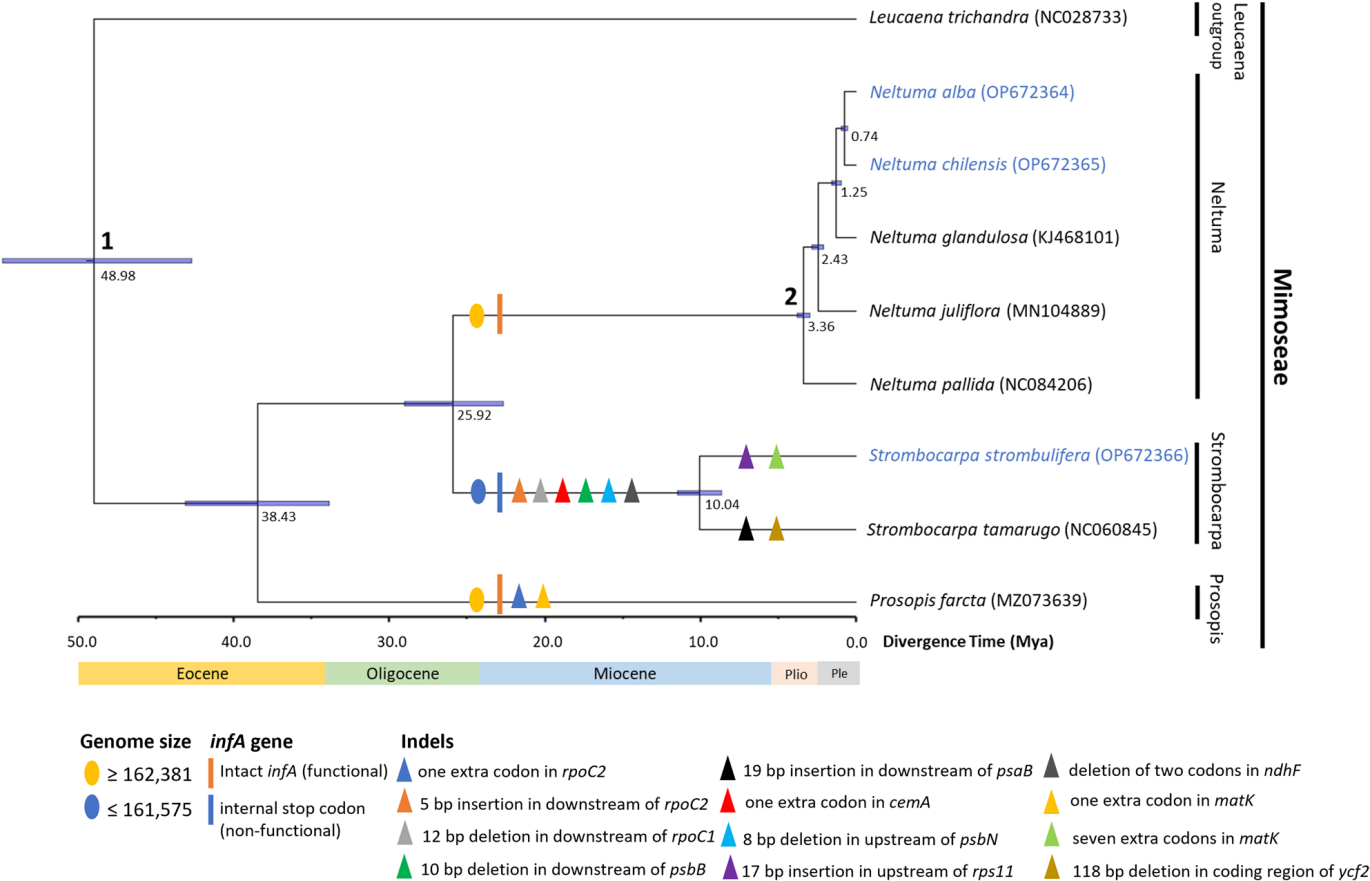
*Neltuma, Strombocarpa and Prosopis* chronogram showing divergence times estimated using BEAST program based on data from nine whole plastid genomes. The divergence times of each clade are displayed near each node. Blue bars represent 95% highest posterior density values for the estimated mean dates. The nodes 1 and 2 correspond to calibration points. Events such as genome size, *infA* gene dysfunctionality and indels of the upstream, downstream and coding regions of all genes are represented in the arms of the phylogenetic tree.

Divergence time for the *Neltuma*, *Strombocarpa* and *Prosopis* species based on the sequence of the plastid genomes is shown in Fig. 7, and suggests that *Netuma*, *Strombocarpa* and *Prosopis* species shared a common ancestor around 48.98 Mya (95% highest posterior density (HPD): 42.69–54.86 Mya) in the Eocene. The age estimate for the split between *Prosopis* in the Old World and the new world species was 38.43 Mya (95% HPD: 33.84–43.08 Mya) in the Eocene. *Strombocarpa* and *Neltuma* genera diverged in the New World around 25.92 Mya (95% HPD: 22.67–29.04 Mya) in the late Oligocene (Fig. 7). Within of the genus *Strombocarpa*, *S. strombulifera* and *S. tamarugo* diverged around 10.04 Mya (95% HPD: 8.65–11.46 Mya) in the late Miocene (Fig. 7). While the species of the genus *Neltuma* diverged into several clades much later, between 3.36 Mya (95% HPD: 2.96–3.77 Mya) and 0.74 Mya (95% HPD: 0.55–0.95 Mya), in the Pliocene (Fig. 7). Taking advantage of our phylogenetic tree with divergence time, we inferred the chronology of genome size, infA gene functionality and indels mutations in *Neltuma*, *Strombocarpa* and *Prosopis*. *P. farcta* gained one codon in both the *rpoC2* and *matK *genes ~ 38.43 Mya ago, which did not happen in *Neltuma* and *Strombocarpa* species. Species of the genus Strombocarpa were predicted to have reduced in plastome size and lost *infA* gene functionality ~ 25.92 Mya ago. A series of indels-type mutation events were found between Neltuma and Strombocarpa in the upstream, downstream and coding regions of the *rpoC2*, *rpoC1*, *psbB*, *psaB*, *cemA*, *psbN*, *rps11*, *ndhF*, *matK* and *ycf2* genes (supplementary information: Fig. S2) which occurred around ~ 25.92 and ~ 10.04 Mya ago (Fig. 7).

## Discussion

Genomic research with NGS technology has developed rapidly, allowing efficient sequencing of complete plastid genomes^56^. Molecular differences in the complete plastid genome between species and individuals provide a good mean of comparison^57,58^. The cp genome offers several advantages over the nuclear genome, such as unique haploid structure, structural conservation, maternal inheritance, and low rate of evolution^57,59^. In our comparative study of the plastid genomes of *N. alba*, *N. chilensis*, and *S. strombulifera* we analyzed gene content, structure, divergence time, and phylogeny and found that the complete plastid genomes of *N. alba* and *N. chilensis* are conserved in size compared to species of the *Strombocarpa* genus*.* The chloroplast of *N. alba* and *N. chilensis* showed similar values for genome size and the number of genes compared to *N. juliflora* and *N. glandulosa*^60^, ~ 163.000 bp for both. The number of genes was similar between the *Neltuma* and *Strombocarpa* genera. Although *S. tamarugo* was thought to have lost the gene *psbL* (remaining with 127 genes only)^24^, we performed a new sequence annotation and finally found the reading frame of the gene. The genome sizes of *S. strombulifera* (160,569 bp) and *S. tamarugo* (161,575 bp) were smaller compared to the *Neltuma* species (~ 163.000 bp)^24^. However, the *Strombocarpa* species presented slightly more GC content (36.0%-36.2%) compared to the *Neltuma* species (35.9%). These GC values fall within the limit of variation registered in others studies^24,60^. Furthermore, a study about several orchid species, showed that the species with a smallest chloroplast size (*Pholidota cantonensis*, 158,786 bp), had a highest GC content (37.47%)^61^, similar to our observations. The plastid genome tends to reduce its size during evolution^62^, and gene length might be affected by selection during the evolution of spermatophytes^63^. The variations in plastid genome size among closely related species can be attributed to IRs, LSC, SSC, intergenic regions, and gene numbers^63^. In this study, very little variation in IRs and intergenic regions was observed between *N. alba* and *N. chilensis*, resulting in very few differences in genome size, while there was a larger variation in these regions in the genomes of *S. strombulifera* and *S. tamarugo* which were < 2500 and < 1500 (respectively) bp smaller than the species from the *Neltuma* genus. Additionally, we found various indels in coding and non-coding regions (explained in more detail further down) that explain the smaller size of the genome of *Strombocarpa* species. Therefore, we assume that *Strombocarpa* species have been exposed to stronger natural selection than *Neltuma* species.

A total of 70 to 100 chloroplast simple sequence repeats (cpSSRs) were founded in the cp genomes of the species of the *Neltuma*, *Strombocarpa* and *Prosopis* genera. Our results showed high variation values in the number of cpSSRs among *Neltuma* and *Strombocarpa* species, being the highest for *N. juliflora* (100) and the lowest for *S. tamarugo* (70). The most abundant cpSSR motif types in *Neltuma*, *Strombocarpa* and *Prosopis* were mono-nucleotides, which is the most abundant repeat type in cp genomes^64,65^. Only *Strombocarpa* species did not show mononucleotide C/G motifs, nor dinucleotide motifs and, additionally, they had a lower number of trinucleotide AAT/ATT motifs. However, the *Strombocarpa* species were the only species that presented the pentanucleotide AATAG/ATTCT motifs. It has been shown in *Cyatheaceae*, that the characteristics of cpSSRs can provide useful phylogenomic information at the genus level, such as phylogenomic relationships, but also about the number, relative abundance, motif type and relative density of cpSSRs^66^. In a similar way, our results demonstrate that the cpSSRs, which are mainly found in introns and intergenic spacers, among *Neltuma* and *Strombocarpa*, both in number and cpSSR motifs, are likely genus specific.

Repeat sequences are considered to play an important role in rearrangements and contain fundamental phylogenomic information^67,68^, although their utility in phylogenetic studies is limited due to homoplasy (i.e. the allele does not always represent common ancestral origin^69^. We found differences in the repeated elements of the cp genome between *Neltuma* and *Strombocarpa* species. The highest total number of repeat elements (palindrome, forward, reverse and complement) was found in *S. tamarugo* (88) and the lowest in *S. strombulifera* (57). In general, the total number of palindromic repeats was less in *Strombocarpa* species than in *Neltuma* species. However, the total number of forward repeats was less in *N. alba* and *N. chilensis* than in the *Strombocarpa* species. On the other hand, the number of complement and reverse (range of 30–39 bp) repeats in *S. tamarugo* was higher than in the *Neltuma* species. In the majority of the species in this study, the most abundant repeat elements detected were, in order: forward, palindromic and reverse. This corresponds to other studies about cp genomes of *Mimosoid* species^70,71^, although *S. tamarugo* is an exception in terms of reverse and complements repeats numbers. These cpSSRs could be used to identify the species studied here. However, it should be taken into consideration that homoplasy might have occurred, so the use of cpSSRs alone for evolutionary studies is limited.

Throughout of the evolution of plastid genomes, structural rearrangements occur, for example in the IRs, which are frequently subject to expansion, contraction or even complete loss^72^. An increased length of IR-SSC boundaries plays an important role in *Mimosoid *plastome size variation^73^. For example, eight *Mimosoid* plastomes of the tribe *Acacia* and *Inga* exhibited an unusual 13 kb IR-SSC boundary shift into the SSC region^71,73^, and the size of these plastomes was found significantly affected by a IR-SC boundary shift, as well as by repeat content^71^. We observed a slight IR expansion into SSC in *S. strombulifera* (26.026 bp) and *S. tamarugo* (25.935 bp). Therefore, the SSC regions of the *Strombocarpa* species showed contraction, and were the shortest SSC regions compared to those of the *Neltuma* and *Prosopis* genera. Asaf et al.^60^ did not detect IR expansion in *Neltuma* and *Prosopis* species, however, they detected a slight expansion in the outgroup species of the genus *Adenanthera* (with a length of 26,028 bp), similar to what we found the in *Strombocarpa* species. The study of Asaf et al.^60^ did not, however, include *Strombocarpa* species to compare to the *Neltuma* and *Prosopis* species. Similar to Asaf^60^, we found a partially duplicated *rps19* gene at the beginnings and ends of the IR regions in *N. alba*, *N. chilensis*, *S. strombulifera* and *S. tamarugo* (including 91 bp in IR). In of most *Mimosoideae* species, the *rps19* is located in the LSC/IRB junction (JLB), with 98–109 bp of the 5′ end of this gene into the IR region^71^. The *ndhF* gene was located closer to the IRB-SSC border (JSB) in *Strombocarpa* species (up to 67 bp) than in *Neltuma* and *Prosopis* species (137 to 156 bp). Likewise, the *ndhF* gene in the species of the genera *Adenanthera*, *Parkia*, *Piptadenia*, *Leucaena* and *Dichrostachys* (*Mimosoideae*) was found entirely within the SSC region (ranging 11 to 150 away from the JSB junction), however, in species of the tribe *Acacia* and *Inga* (*Mimosoideae*) it was found within the JSB junction, resulting in the duplication of this gene^71^. Several models concerning the expansion and contraction of IR regions have been proposed to explain the possible mechanisms that result in shifts in the IR-LSC junctions^74^. In our case, we detected that *Strombocarpa* species had a larger contraction of the LSC region then *Neltuma* and *Prosopis* species. The structural differences presented among the plastomes of the *Neltuma* and *Strombocarpa* species reinforce the idea and necessity to disintegrate the *Prosopis* cluster, as proposed by Hughes et al.^4^. However, for the new genera it would have been recommendable to have kept the names of the sections *Algarobia* and *Strombocarpa*, as proposed by Burkart^6^ for the new genera. Alternatively, there are studies that justify maintaining the genus *Prosopis* instead of disintegrating it, due to the segregation percentages shown in spineless *Prosopis* versus spiny *Prosopis*^75^. The authors explain that the lack of spines would be controlled by two recessive genes, and that two genes should not be enough to place taxa in different genera^75^.

Among the Neltuma species, the divergence distance between *N. alba* and *N. chilensis* was the lowest divergence observed. According to the results obtained using p-distance, there is a high sequence divergence in plastid genomes between the genera *Neltuma* and *Strombocarpa*. In other studies, a large variation was also observed when using p-distance for chloroplast genomes of Styrax genus trees, ranging from 0.0003 to 0.00611^76^. The p-distance results revealed that there is very low evolutionary divergence within the genus *Neltuma* (0.00100), whereas the evolutionary divergence between *Strombocarpa* species was much higher (0.00522). However, within the tribe *Mimoseae* the distance between *Neltuma* and *Strombocarpa* species was one of the lowest, which much larger distances (up to 0.0853) to other species of the tribe. Our results both explain why *Neltuma* and *Strombocarpa* until recently were clustered in the *Prosopis* genus, as they are still very similar when compared to other species of the tribe, but also why they are now split into 3 different genera, as the divergence distances between the 3 genera is much larger than within these genera.

The phylogenomic results (ML and BI) based on 76 protein-coding genes of the plastid genome of nine *Mimosoideae* species showed that *S. strombulifera* formed a strongly supported group with *S. tamarugo* (BP = 100; PP = 1.00), and the *Neltuma* group appeared paraphyletic because *P. cineraria* was part of a well-supported clade (BP = 62; PP = 1.00) with *N. juliflora*, *N. alba* and *N. chilensis*. *P. farcta*, however appeared as sister group of *Neltuma* and *Strombocarpa* clade, as expected. Within the *Neltuma* clade, *N. alba* formed a highly supported clade with *N. chilensis* (BP = 100; PP = 1.00), and so did *N. juliflora* with *P. cineraria* (BP = 100; PP = 1.00), whereas *N. glandulosa* appeared as a strongly supported sister group to both (BP = 100; PP = 1.00). With the exception of *P. cineraria* (further discussed in the next paragraph), the *Neltuma* group was monophyletic with *Strombocarpa* group as its sister clade. Although *S. strombulifera* and *S. tamarugo* formed a well-supported group, these two species showed important differences in genome size, number of genes and genetic divergence with high degree of variation. These genetic differences in the chloroplast correspond to the findings of Burkart^6^ who separated *S. tamarugo* and *S. strombulifera* into the *Cavernicarpae* and *Strombocarpa*e series, respectively. The same was observed by Catalano et al.^13^ through a three-marker analysis (*trnS-psbC*, *G3pdh*, *NIA*), who found two well supported groups, one of them corresponding to the *Cavenicarpae* series (including *Prosopis ferox* and *P. tamarugo*) and the other formed by North American species of the *Strombocarpa*e series (including *Prosopis pubescens* and *Prosopis palmeri*).

Undoubtedly, the biggest inconsistency observed in our phylogenomic analysis was the nesting of *P. cineraria* within the *Neltuma* clade. According to the results of Asaf et al.^60^, *P. cineraria* forms a group with high support with *N. juliflora*. It is interesting and unexpected that *P. cineraria* did not form a group with *P. farcta*, both of them being Old World species, but nested with the New World species *N. juliflora*, *N. glandulosa*, *N. alba* and *N. chilensis* instead. However, according to the phylogenomic analysis performed by Catalano et al.^13^, there are more distant relationships among species from the Old World sections and closer relationships among species of the American sections (*Strombocarpa*, *Algarobia*, and *Monilicarpa* sections). *Prosopis cineraria* is one of the most common trees of the Indian desert, Arabian Peninsula and, in general, is abundant throughout the middle east^60,77^, whereas *N. juliflora* is native to the Caribbean, Central and northern South America^78^. However, *Neltuma juliflora* was introduced to Ethiopia and the Middle East around 1970 and over the years this species has spread outside the plantation areas, adversely affecting natural habitats and rangelands^79^. This invasive plant is characterized by vigorous growth which helps it to outcompete indigenous plant species^80^. *Neltuma juliflora* seeds survive in livestock and warthogs’ droppings, which serve as a vehicle for the plant to reach distant areas and to expand their distribution throughout the region^80,81^. We hypothesize that *N. juliflora* might have crossed with some individuals of *P. cineraria* in a natural way, giving offspring to a hybrid with a phenotype resembling *P. cineraria* but, when *N. juliflora* acted as the maternal part, with the plastid genome of *N. juliflora*. This could be a logic explanation for the nesting of *P. cineraria* within the *Neltuma* clade, if the samples used by Asaf et al.^60^ were obtained from a *P. cineraria* resembling hybrid.

Estimate of divergence time in plant groups have been important in order to understand their phylogeographic history and evolutionary biology^82^. Due to the inconsistencies observed in the placement of *P. cineraria* among the other species of *Mimoseae* in the phylogenetic tree, we decided not to consider this species for the estimation of divergence time. Our molecular dating analysis suggests that *Leucaena trichandra* as root species diverged in the Early Eocene (mean = 48.98 Mya; 95% HPD = 42.69–54.86 Mya). Later, *P. farcta* diverged in the Middle Eocene (mean = 38.43 Mya; 95% HPD = 33.84–43.08 Mya). Our results coincide with a previous study, which indicates that the divergence between *Strombocarpa* and *Neltuma* genera occurred in the Oligocene^13^ (mean = 25.92 Mya; 95% HPD = 22.67–29.04 Mya). The molecular divergence time found in *Neltuma* and *Strombocarpa* genera is relatively close to the diversification of the major clades in the subfamily *Mimosoideae*, which occurred in the Late Miocene^13,49^. Our results showed that *Strombocarpa* diverged in the Late Miocene (mean = 10.04 Mya; 95% HPD = 8.65–11.46 Mya), which is supported by the fossil *Prosopisinoxylon anciborae*, a *Mimosoideae* species with a high similarity to genus *Prosopis* L. (currently re-delimitated), reported to have occurred during the Late Miocene in the Catamarca Province, Argentina^83^. Additionally, a similar divergence time, around 9.21 Mya (8.35–10.07), for the genus *Strombocarpa* was found Catalano et al.^13^. Our results also showed that the *Neltuma* genus started diverging in the Pliocene (mean = 3.36 Mya; 95% HPD = 2.96–3.77 Mya) and continued in the Pleistocene. This corresponds to the Series *Pallidae*, *Chilenses*, and *Ruscifoliae* species (e.g. *N. alba*, *N. juliflora*, *N. glandulosa*, *N. chilensis*, *N. alpataco* and *N. nigra*) whose divergence time started in the Pliocene and continued in the Pleistocene, (mean = 3.65 Mya; 95% HPD = 3.31–3.99 Mya)^13^. Combined with the divergence time data, our phylogenetic tree allows us to infer the chronology of genome size, *infA* gene functionality and indel mutations in the plastid genome of *Neltuma* and *Strombocarpa*. Part of the moderate reduction in genome size in *Strombocarpa* species can be attributed to rearrangements in the SSC, LSC and IRs, as discussed in the previous paragraph. Other reductions occurred due to a moderate amount of indels located in coding regions, upstream and downstream regions of several genes, but we also found several deletions in intergenic regions in *Strombocarpa* species (data not shown). Another interesting, but not uncommon, find was the presence of a stop codon in the *infA* gene in *Strombocarpa* species, interrupting the translation of its hypothetical protein. This has been shown for *Veratrum* species as well, cataloguing the *InfA* gene as pseudogene^84^. In fact, the *infA* gene is considered one of the most frequently lost genes in angiosperms, and it is believed that its functional copy has been transferred to the nucleus^85^.

Tree species such as *Neltuma* and *Strombocarpa* species are subject to a number of ecological selective pressures due to the hostile conditions of the Atacama Desert. Chloroplast genes are involved in regulatory responses to various abiotic stresses, including heat, chilling, salinity, drought and radiation^86,87^. Therefore, the here presented plastid genomes of the *Neltuma* and *Strombocarpa* species can play an important role in understanding the plants adaptations to these hostile environments.

The plastid genome structure of legumes is particularly interesting, because it contains multiple rearrangements, expansions, contractions, and loss of genetic content, which are all very useful for phylogenomic studies^87^. Phylogenomic analysis can aid conservation of species through the confirmation of taxonomic status, clarification of evolutionary relationships and consequently the determination for conservation priorities^88^. Additionally, phylogeographic studies offer valuable information for conservation purposes as they describe the geographical distribution of genetic variability, and therefore the genetic health among species populations^89^. With this study, we discovered differences in plastid genomes of *Neltuma* and *Strombocarpa* species improving our understanding of their phylogeny and evolution. This information can be used to identify the distinct species in the communities of these valuable species. Which in turn can help management strategies, e.g. exchange of pollen between populations, to increase their genetic variability before it is too late and they disappear.

## Conclusion

In this work, we present for the first time the assembly and characterization of the plastid genomes of *Neltuma alba*, *Neltuma chilensis* and *Strombocarpa strombulifera*. The chloroplasts presented in this study provide a better understanding of the diversification of *Neltuma*, *Strombocarpa* and *Prosopis* as well as important information for evolutionary, phylogenomic and biogeographic studies for other species of the *Fabaceae* family. We found enough variation in genome size, GC content, indels, repetitive elements and divergence to support the disintegration of the former genus *Prosopis* L.

### Supplementary Information

Supplementary Figure S1.

Supplementary Figure S2.

Supplementary Tables.

## Data Availability

The datasets generated and analyzed during the current study are available in the Genome Database on National Center for Biotechnology Information (NCBI) repository under the accession number OP672364 for Neltuma alba, OP672365 for Neltuma chilensis and OP672366 for Strombocarpa strombulifera. The BioProject and BioSample accession numbers on NCBI for Neltuma alba are PRJNA1026123 and SAMN37734720, for Neltuma chilensis are PRJNA1026131 and SAMN37735133, and for Strombocarpa strombulifera are PRJNA1026137 and SAMN37735326. The identification of the plant material was carried out by Roberto Contreras-Díaz, according to the keys described by Burkart (1976). It was also confirmed by CONAF professionals and recognized by the expert Nicolás García of the Universidad de Chile.

## Code availability

The sequence data is available in GenBank with the codes OP672364, OP672365, OP672366.
