# Supplementary figures and images for: Structure, gene composition, divergence time and phylogeny analysis of the woody desert species Neltuma alba, Neltuma chilensis and Strombocarpa strombulifera
Source: Sci Rep. 2024 Jun 13;14:13604. doi: 10.1038/s41598-024-64287-y (PMC11176173; doi:10.1038/s41598-024-64287-y)

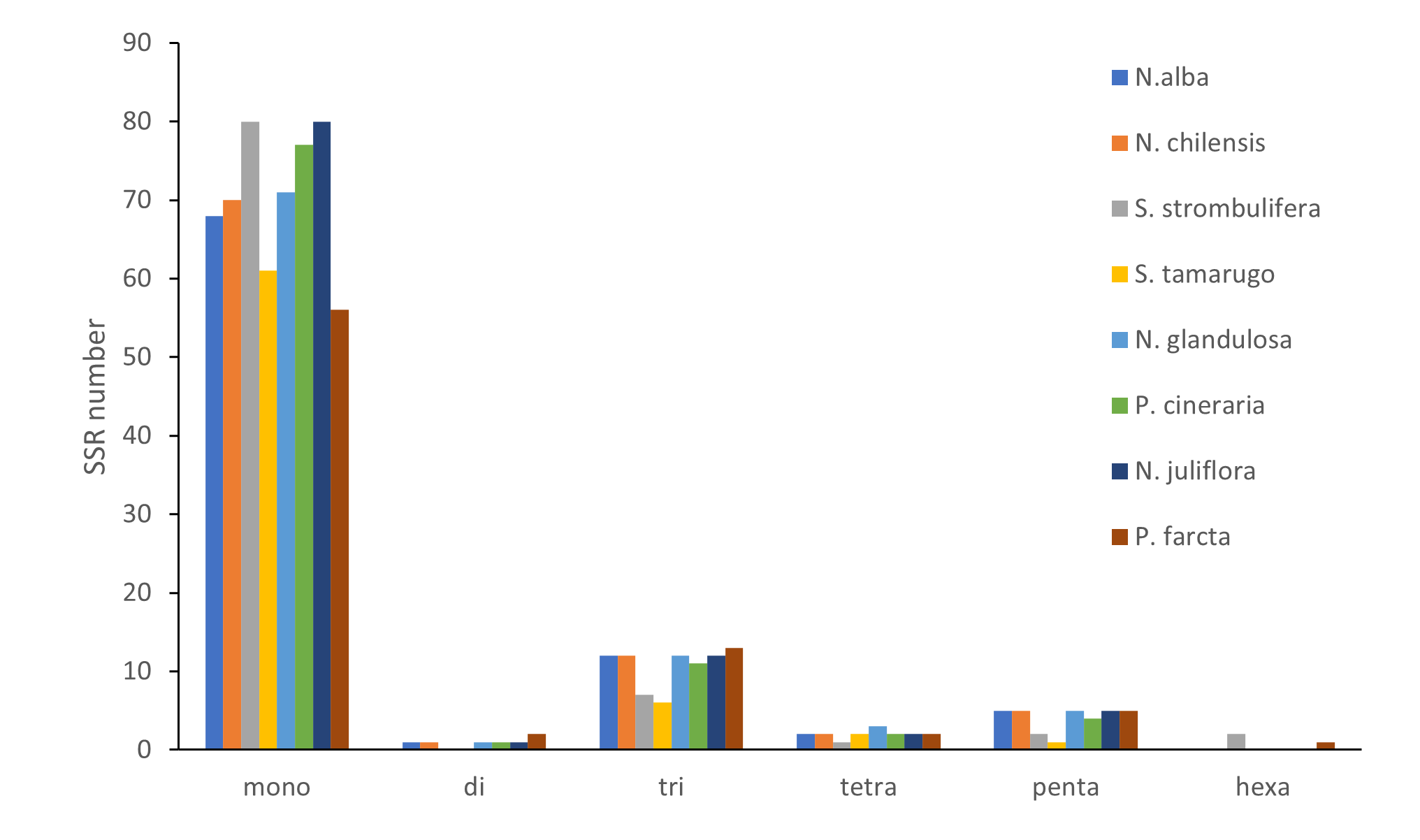

Supplement: Supplementary file 1 — Supplementary Figure S1. [file 41598_2024_64287_MOESM1_ESM.tif]
