# Supplementary material for: Structure, gene composition, divergence time and phylogeny analysis of the woody desert species Neltuma alba, Neltuma chilensis and Strombocarpa strombulifera
Source: Sci Rep. 2024 Jun 13;14:13604. doi: 10.1038/s41598-024-64287-y (PMC11176173; doi:10.1038/s41598-024-64287-y)

**Figure S2**. Indels found in the upstream, downstream and coding regions of genes of Neltuma, Strombocarpa and Prosopis species

**A**. One additional triplet (codon) in the coding region of the *rpoC2* gene in *Prosopis farcta*


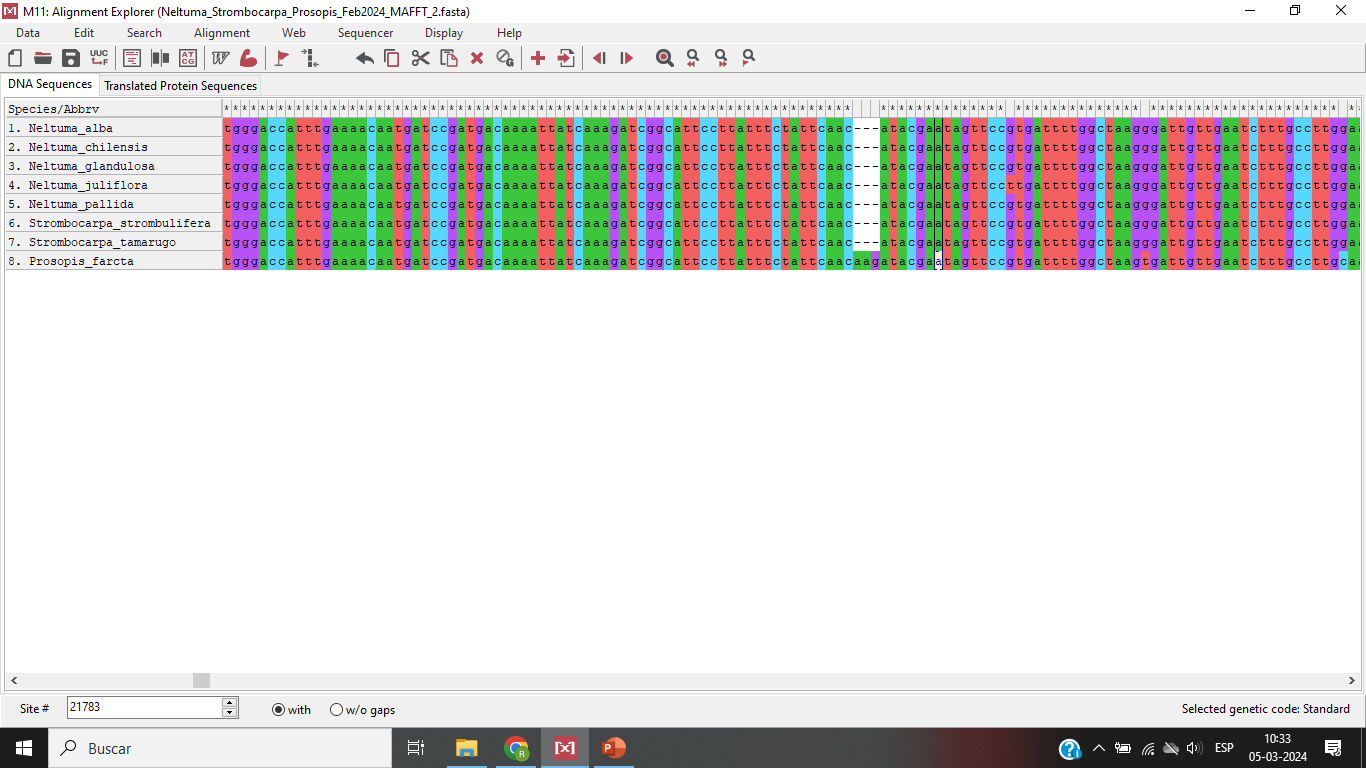


**B**. 5 bp insertion in the downstream of the *rpoC2* gene in *S. strombulifera* and *S. tamarugo*.


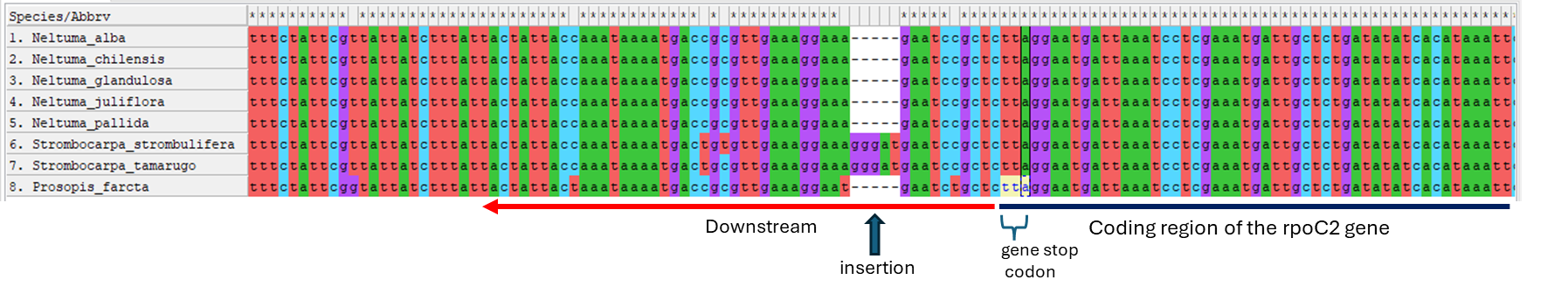


**C**. 12 bp deletion in the downstream region of the *rpoC1* gene in *S. strombulifera* and *S. tamarugo*.


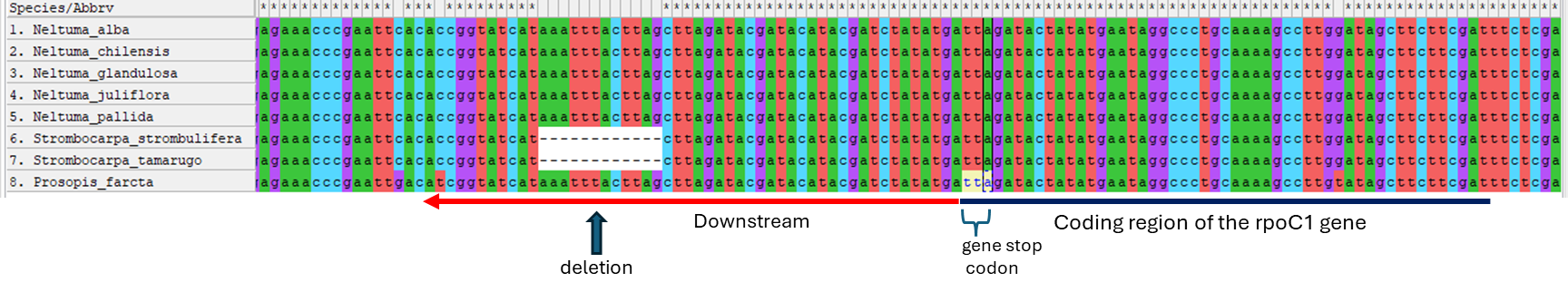


**D**. 19 bp insertion in the downstream region of the *psaB* gene in *S. tamarugo*.


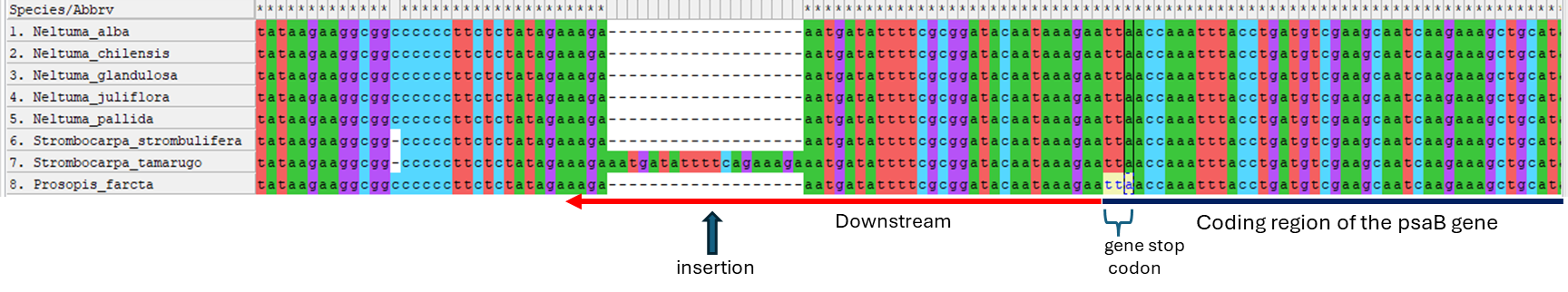


**E**. One additional triplet (codon) in the coding region of the *cemA* gene in *S. strombulifera* and *S. tamarugo.*


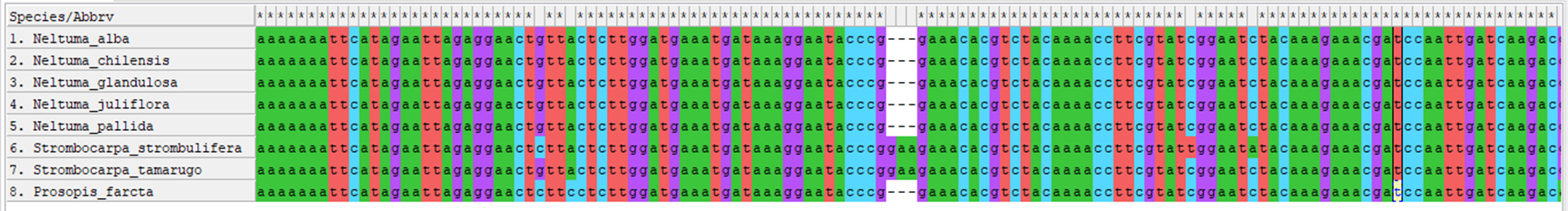


**F**. 10 bp deletion in the downstream region of the *psbB* gene in *S. strombulifera* and *S. tamarugo*.


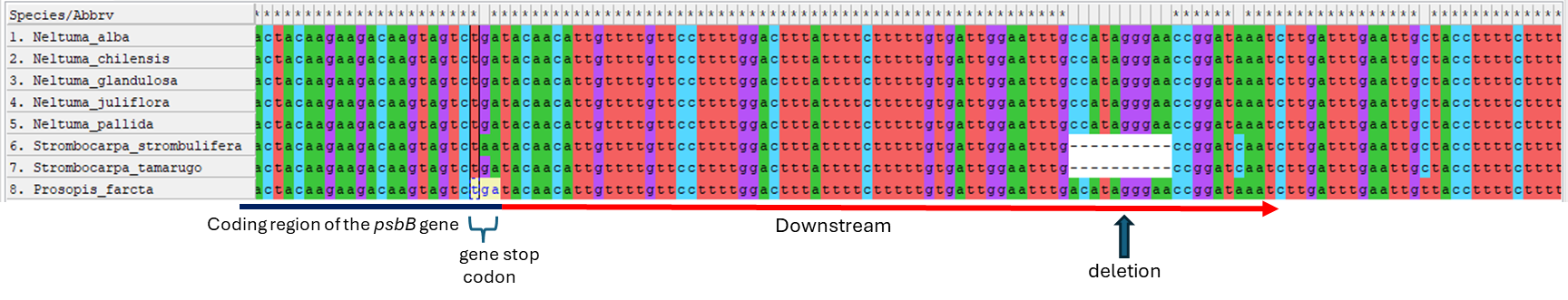


**G**. 8 bp deletion in the upstream region of the *psbN* gene in *S. strombulifera* and *S. tamarugo*.


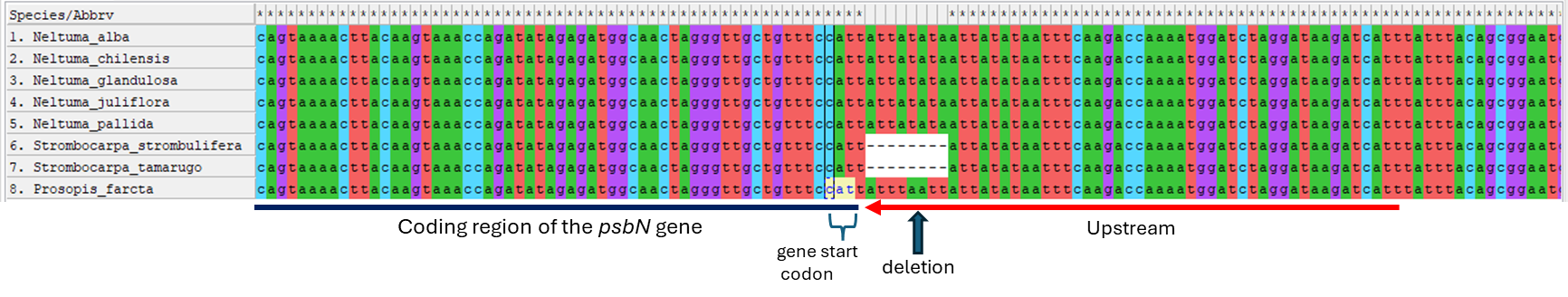


**H**. 17 bp insertion in the upstream region of the *rps11* gene in *S. strombulifera*.


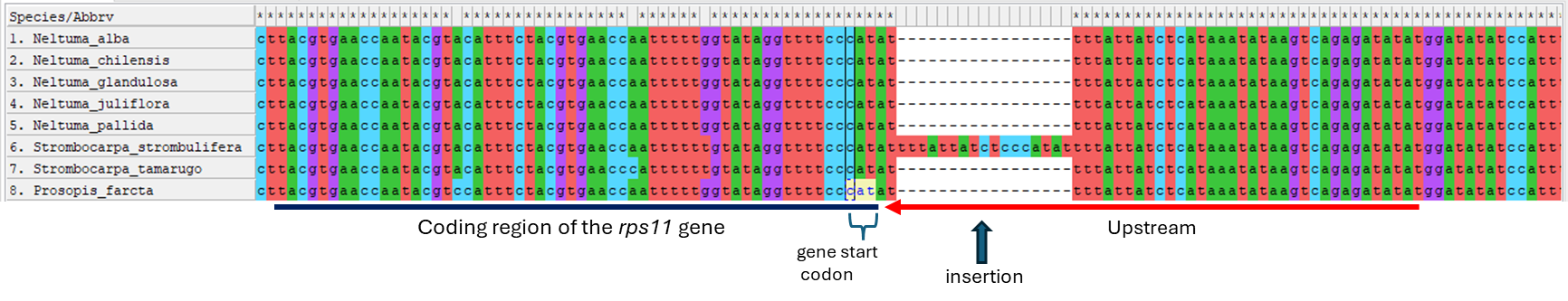


**I**. Deletion of two triplets (codon) in the coding region of the *ndhF* gene in *S. strombulifera* and *S. tamarugo*.


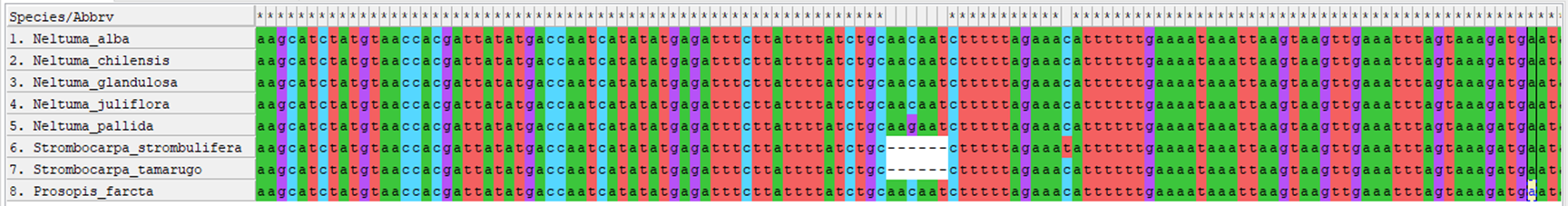


**J**. Additional triplet (codon) in the coding region of the *matK* gene in *Prosopis farcta*


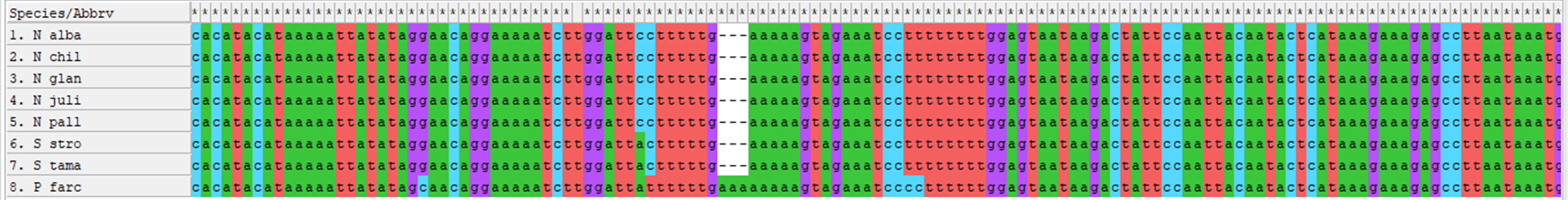


**K**. Addition of seven triplets (codon) in the coding region of the *matK* gene in *S. strombulifera*.


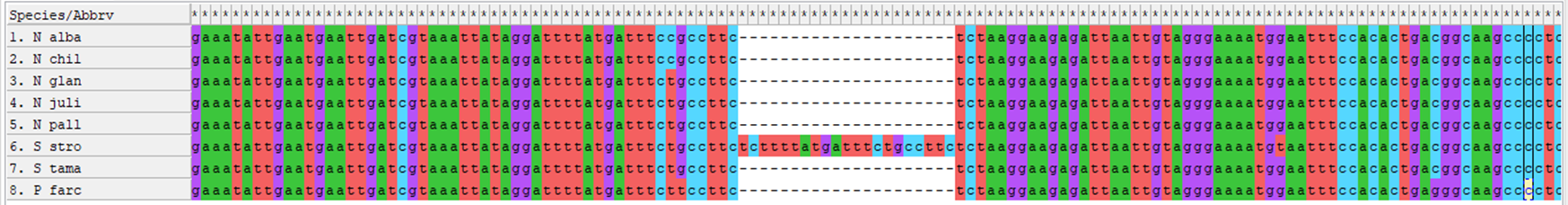


**L**. 118 bp deletion in the coding region of the *ycf2* gene in *S. tamarugo*.


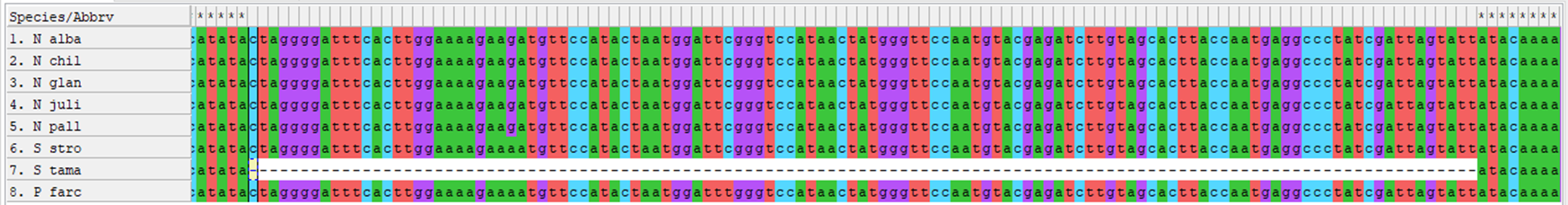

Supplement: Supplementary file 2 — Supplementary Figure S2. [file 41598_2024_64287_MOESM2_ESM.docx]
