# Supplementary material for: Structure, gene composition, divergence time and phylogeny analysis of the woody desert species Neltuma alba, Neltuma chilensis and Strombocarpa strombulifera
Source: Sci Rep. 2024 Jun 13;14:13604. doi: 10.1038/s41598-024-64287-y (PMC11176173; doi:10.1038/s41598-024-64287-y)
Supplement: Supplementary file 3 — Supplementary Tables. [file 41598_2024_64287_MOESM3_ESM.docx]

**Table S1**. The p-distance value among eight plastid genomes of Neltuma, Strombocarpa and Prosopis species.

| Species | *N. alba* | *N. chilensis* | *N. glandulosa* | *N. juliflora* | *N. pallida* | *S. strombulifera* | *S. tamarugo* | *P. farcta* |
| --- | --- | --- | --- | --- | --- | --- | --- | --- |
| *N. alba* | 0.000000 |  |  |  |  |  |  |  |
| *N. chilensis* | 0.000368 | 0.000000 |  |  |  |  |  |  |
| *N. glandulosa* | 0.000412 | 0.000295 | 0.000000 |  |  |  |  |  |
| *N. juliflora* | 0.001321 | 0.001203 | 0.001012 | 0.000000 |  |  |  |  |
| *N. pallida* | 0.001290 | 0.001185 | 0.001043 | 0.001919 | 0.000000 |  |  |  |
| *S. strombulifera* | 0.011758 | 0.011656 | 0.011527 | 0.012356 | 0.011828 | 0.000000 |  |  |
| *S. tamarugo* | 0.009586 | 0.009471 | 0.009355 | 0.010162 | 0.009739 | 0.005225 | 0.000000 |  |
| *P. farcta* | 0.015620 | 0.015502 | 0.015286 | 0.016211 | 0.015604 | 0.020162 | 0.018099 | 0.000000 |

**Table S2**. The p-distance value among twenty plastid genomes of tribe Mimoseae species.

| Species | 1 | 2 | 3 | 4 | 5 | 6 | 7 | 8 | 9 | 10 | 11 | 12 | 13 | 14 | 15 | 16 | 17 | 18 | 19 | 20 |
| --- | --- | --- | --- | --- | --- | --- | --- | --- | --- | --- | --- | --- | --- | --- | --- | --- | --- | --- | --- | --- |
| *1.-N. alba* | 0.000000 |  |  |  |  |  |  |  |  |  |  |  |  |  |  |  |  |  |  |  |
| *2.-N. chilensis* | 0.000368 | 0.000000 |  |  |  |  |  |  |  |  |  |  |  |  |  |  |  |  |  |  |
| *3.-N. glandulosa* | 0.000412 | 0.000295 | 0.000000 |  |  |  |  |  |  |  |  |  |  |  |  |  |  |  |  |  |
| *4.-N. juliflora* | 0.001321 | 0.001203 | 0.001000 | 0.000000 |  |  |  |  |  |  |  |  |  |  |  |  |  |  |  |  |
| *5.-N. pallida* | 0.001290 | 0.001185 | 0.001037 | 0.001913 | 0.000000 |  |  |  |  |  |  |  |  |  |  |  |  |  |  |  |
| *6.-S. strombulifera* | 0.011777 | 0.011674 | 0.011565 | 0.012388 | 0.011834 | 0.000000 |  |  |  |  |  |  |  |  |  |  |  |  |  |  |
| *7.-S. tamarugo* | 0.009592 | 0.009478 | 0.009381 | 0.010181 | 0.009733 | 0.005225 | 0.000000 |  |  |  |  |  |  |  |  |  |  |  |  |  |
| *8.-P. farcta* | 0.015626 | 0.015508 | 0.015293 | 0.016206 | 0.015610 | 0.020224 | 0.018142 | 0.000000 |  |  |  |  |  |  |  |  |  |  |  |  |
| *9.-C. gabunensis* | 0.016878 | 0.016719 | 0.016602 | 0.017380 | 0.016569 | 0.016919 | 0.014807 | 0.019748 | 0.000000 |  |  |  |  |  |  |  |  |  |  |  |
| *10.-L. trichandra* | 0.020998 | 0.020912 | 0.020798 | 0.021556 | 0.020432 | 0.021076 | 0.019244 | 0.025264 | 0.017680 | 0.000000 |  |  |  |  |  |  |  |  |  |  |
| *11.-P. javanica* | 0.017261 | 0.017103 | 0.017021 | 0.017740 | 0.017116 | 0.017495 | 0.015274 | 0.021274 | 0.014380 | 0.018035 | 0.000000 |  |  |  |  |  |  |  |  |  |
| *12.-P. communis* | 0.018792 | 0.018690 | 0.018578 | 0.019349 | 0.018504 | 0.018675 | 0.016718 | 0.022673 | 0.016051 | 0.020435 | 0.014990 | 0.000000 |  |  |  |  |  |  |  |  |
| *13.-S. adstringens* | 0.019639 | 0.019536 | 0.019410 | 0.020189 | 0.019342 | 0.019963 | 0.017889 | 0.023340 | 0.017148 | 0.021343 | 0.015440 | 0.010990 | 0.000000 |  |  |  |  |  |  |  |
| *14.-P. africanum* | 0.024453 | 0.024447 | 0.024377 | 0.025106 | 0.024195 | 0.024669 | 0.022768 | 0.027094 | 0.020015 | 0.026992 | 0.022110 | 0.023408 | 0.024193 | 0.000000 |  |  |  |  |  |  |
| *15.-A. microsperma* | 0.025474 | 0.025449 | 0.025331 | 0.026033 | 0.025004 | 0.025582 | 0.023739 | 0.028086 | 0.021402 | 0.027295 | 0.022965 | 0.024537 | 0.025270 | 0.019653 | 0.000000 |  |  |  |  |  |
| *16.-D. cinerea* | 0.024398 | 0.024372 | 0.024218 | 0.025014 | 0.024243 | 0.024562 | 0.023297 | 0.027521 | 0.022122 | 0.023453 | 0.022317 | 0.024147 | 0.024647 | 0.028492 | 0.029795 | 0.000000 |  |  |  |  |
| *17.-M. diplotricha* | 0.030628 | 0.030513 | 0.030428 | 0.031103 | 0.030367 | 0.030775 | 0.029258 | 0.034442 | 0.028597 | 0.031946 | 0.026884 | 0.026875 | 0.027609 | 0.035019 | 0.035870 | 0.035389 | 0.000000 |  |  |  |
| *18.-M. pigra* | 0.034414 | 0.034375 | 0.034293 | 0.034968 | 0.034028 | 0.034314 | 0.032779 | 0.038178 | 0.032346 | 0.036088 | 0.030513 | 0.030440 | 0.031046 | 0.038338 | 0.039387 | 0.038769 | 0.015027 | 0.000000 |  |  |
| *19.-X. xylocarpa* | 0.037094 | 0.037003 | 0.036889 | 0.037616 | 0.036675 | 0.037269 | 0.035453 | 0.039811 | 0.032886 | 0.038446 | 0.034806 | 0.036254 | 0.036968 | 0.032598 | 0.029671 | 0.041563 | 0.046372 | 0.049615 | 0.000000 |  |
| *20.-**E. phaseoloides* | 0.073068 | 0.072979 | 0.072856 | 0.073516 | 0.072186 | 0.072261 | 0.071309 | 0.075311 | 0.069556 | 0.074744 | 0.071360 | 0.072197 | 0.072906 | 0.067710 | 0.068984 | 0.076573 | 0.082479 | 0.085305 | 0.080203 | 0.000000 |
